# Identification of a gustatory receptor tuned to sinigrin in the cabbage butterfly *Pieris rapae*

**DOI:** 10.1371/journal.pgen.1009527

**Published:** 2021-07-15

**Authors:** Jun Yang, Hao Guo, Nan-Ji Jiang, Rui Tang, Guo-Cheng Li, Ling-Qiao Huang, Joop J. A. van Loon, Chen-Zhu Wang

**Affiliations:** 1 State Key Laboratory of Integrated Management of Pest Insects and Rodents, Institute of Zoology, Chinese Academy of Sciences, Beijing, China; 2 CAS Center for Excellence in Biotic Interactions, University of Chinese Academy of Sciences, Beijing, China; 3 Laboratory of Entomology, Plant Sciences Group, Wageningen University and Research, Wageningen, the Netherlands; University of Kentucky, UNITED STATES

## Abstract

Glucosinolates are token stimuli in host selection of many crucifer specialist insects, but the underlying molecular basis for host selection in these insects remains enigmatic. Using a combination of behavioral, electrophysiological, and molecular methods, we investigate glucosinolate receptors in the cabbage butterfly *Pieris rapae*. Sinigrin, as a potent feeding stimulant, elicited activity in larval maxillary lateral sensilla styloconica, as well as in adult medial tarsal sensilla. Two *P*. *rapae* gustatory receptor genes *PrapGr28* and *PrapGr15* were identified with high expression in female tarsi, and the subsequent functional analyses showed that *Xenopus* oocytes only expressing *PrapGr28* had specific responses to sinigrin; when ectopically expressed in *Drosophila* sugar sensing neurons, PrapGr28 conferred sinigrin sensitivity to these neurons. RNA interference experiments further showed that knockdown of *PrapGr28* reduced the sensitivity of adult medial tarsal sensilla to sinigrin. Taken together, we conclude that PrapGr28 is a gustatory receptor tuned to sinigrin in *P*. *rapae*, which paves the way for revealing the molecular basis of the relationships between crucifer plants and their specialist insects.

## Author summary

Preference of crucifer specialist insects to glucosinolates is well known in the field of insect-plant interactions, but its molecular basis is unclear. This study uses an integrative approach to investigate the molecular basis of glucosinolate detection by gustatory receptor neurons in the larval mouthparts and adult forelegs of the cabbage butterfly *Pieris rapae*, and finally reveal that PrapGr28 is a bitter receptor tuned to sinigrin. The current work takes a significant step towards identifying gustatory receptors tuned to glucosinolates, crucial recognition signals in crucifer host plants, providing insights into co-evolution of herbivorous insects and their host plants.

## Introduction

Plant secondary compounds play a central role in co-evolution between herbivorous insects and plants [1,2]. Most of them act as defensive chemicals against attack by herbivorous insects through inhibiting feeding and oviposition. To counteract plant chemical defenses, herbivores have developed multiple adaptations through avoidance, detoxification, and selective storage. An intriguing adaptation is found in some monophagous and oligophagous insects that even use these compounds as token stimuli to recognize host plants for feeding or oviposition [3,4].

Glucosinolates comprise a group of important secondary compounds in the plant family Cruciferae (Brassicaceae), and have been generally considered to have a defensive function against generalist herbivores [5–8]. According to the side chain of the precursor amino acid, they are divided into three classes, aliphatic, indolic, and aromatic glucosinolates [9,10]. Among common glucosinolates, sinigrin, glucoraphanin and gluconapin are aliphatic, glucobrassicin and neoglucobrassicin are indolic, and gluconasturtiin is aromatic. It has long been known that glucosinolates act as token stimuli, meaning a stimulus indispensable to trigger a behavioral response, for feeding and oviposition in crucifer specialist herbivores. The token stimulus function was first demonstrated in two *Pieris* butterfly species, *Pieris brassicae* and *P*. *rapae*. Sinigrin, glucotropaeolin, glucocapparin and glucomoringin stimulate larval feeding of *P*. *brassicae* [11–13]. Sinigrin, glucosinalbin (sinalbin), glucotropaeolin and glucobrassicin stimulate oviposition by *P*. *brassicae* [14,15]. For *P*. *rapae*, it has been reported that sinigrin and gluconasturtiin significantly contribute to elicit larval feeding [16,17]. Ten glucosinolates differentially stimulate oviposition by *P*. *rapae* females: glucobrassicin and gluconasturtiin have high stimulatory activity, followed by glucocapparin, glucosinalbin, glucotropaeolin, sinigrin and glucoalyssin, while glucocheirolin, glucoerucin and glucoiberin show weak activity [18,19]. In addition, glucosinolates also stimulate feeding and/or oviposition by the diamondback moth *Plutella xylostella*, the leaf beetle *Phyllotreta cruciferae*, the cabbage aphid *Brevicoryne brassicae*, and the turnip sawfly *Athalia rosae* among others [13,20–23]. These studies show that glucosinolates are important token stimuli for host recognition in crucifer specialist herbivores belonging to four insect orders.

Stimulatory effects of glucosinolates on both larval feeding and adult oviposition behavior are mediated by the gustatory receptor neurons (GRNs) in taste sensilla of *Pieris* butterflies [17,19,24,25]. Taste sensilla are mainly distributed on the mouthparts in larvae, and the tarsi, proboscis and antennae in adults [26,27]. The GRNs sensitive to glucosinolates in *Pieris* butterfly larvae have been characterized and occur in two sensilla styloconica on the maxillary galea of larvae [3,25,26,28]. In *P*. *brassicae* larvae, one GRN located in the lateral sensillum styloconicum is sensitive to glucobrassicin, glucocapparin, sinigrin, glucotropaeolin, glucoiberin, and glucosinalbin, and another one located in the medial sensillum styloconicum only responds to the aromatic glucosinalbin and glucotropaeolin, suggesting that these specialised GRNs in two sensilla styloconica show distinct but partially overlapping response profiles to different glucosinolates [25,28]. In *P*. *brassicae* adults, the medial gustatory sensilla on female tarsi were later found to have GRNs with similar response profiles, exhibiting strong responses to glucotropaeolin and sinigrin [14,29]. For *P*. *rapae*, it has been demonstrated that it likewise possesses glucosinolate-sensitive-GRNs in the larval maxillary lateral sensillum styloconicum [17] and the tarsal medial sensilla of female adults [19]. Other crucifer specialist herbivores, such as *Pl*. *xylostella*, and *Pieris napi* [24,26], also harbour GRNs responding to glucosinolates. However, it is worth pointing out that although the GRNs sensitive to glucosinolates have been characterized in various insect species, nothing is known about the gustatory receptors (GRs) expressed in the dendrites of these GRNs, which hinder a thorough understanding of the interactions and co-evolution between crucifer specialist insects and their host-plants.

Most of the current knowledge about insect GRs comes from studies of the fruit fly *Drosophila melanogaster*. Insect GRs are seven transmembrane domain proteins with an intracellular N-terminus and an extracellular C-terminus [30]. They can be divided into three main classes: carbon dioxide (CO_2_), sugar and ‘bitter’ receptors [30]. ‘Bitter’ is a generic term derived from the sensation elicited in humans by tasting plant allelochemicals such as caffeine, cucurbitacin *etc* [31]. In plant-feeding insects, ‘bitter’ receptors are the GRs that play a key role in recognition of plant allelochemicals, which are usually deterrents with an inhibitory effect on insect feeding and oviposition [3,5–7,25]. Among insects, only in *D*. *melanogaster* have bitter GRs been studied in detail. Six bitter receptors of *Drosophila* have been identified as commonly expressed receptors: Gr32a, Gr33a, Gr39a.a, Gr66a, Gr89a and Gr93a [32,33]. They can form different GR complexes with specific GRs to detect certain bitter compounds, such as quinine, sparteine, escin, denatonium, berberine, lobeline, theobromine, saponin, DEET, coumarin, theophylline, umbelliferone, caffeine and so on [32–35]. However, no receptor has been found for glucosinolates in *Drosophila* studies.

With the advent of the next-generation of genome and transcriptome sequencing, a large number of bitter GR genes have been identified in various species in the order Lepidoptera [36–38], but only a few bitter receptors have been functionally characterized. In the swallowtail butterfly *Papilio xuthus*, a bitter receptor, PxutGr1, responds to synephrine, which is involved in the oviposition site recognition by this species [39]. This means that ‘bitter’ receptors do not necessarily encode inhibitory bitter substances, they can encode stimulatory substances such as token stimuli as well. In the silkworm *Bombyx mori*, both BmGr16 and BmGr18 are activated by coumarin and caffeine, and BmGr53 is widely tuned to coumarin, caffeine and pilocarpine [40]. In addition, BmGr66 is a major factor affecting feeding preference, but its ligand remains unknown [41]. In *P*. *rapae*, five sugar receptors and three bitter receptors have been identified by using genome sequencing [42], but their functions have not been characterized. In particular, the repertoire of GRs detecting glucosinolates is unknown.

In order to reveal the molecular mechanism of taste perception of glucosinolates in *P*. *rapae*, we first tested how different glucosinolates affect larval feeding, and then characterized the GRNs sensitive to glucosinolates in both larval mouthparts and adult tarsi. Next, we identified two bitter receptors highly expressed in the female tarsi via transcriptome sequencing and quantitative real-time PCR (qRT-PCR) analysis, and then analyzed their function by using two heterologous expression systems and RNA inference (RNAi). We finally demonstrate that *PrapGr28* is a gene coding for the receptor tuned to sinigrin.

## Results

### Feeding stimulation by glucosinolates in *P*. *rapae* larvae

**Fig 1:**
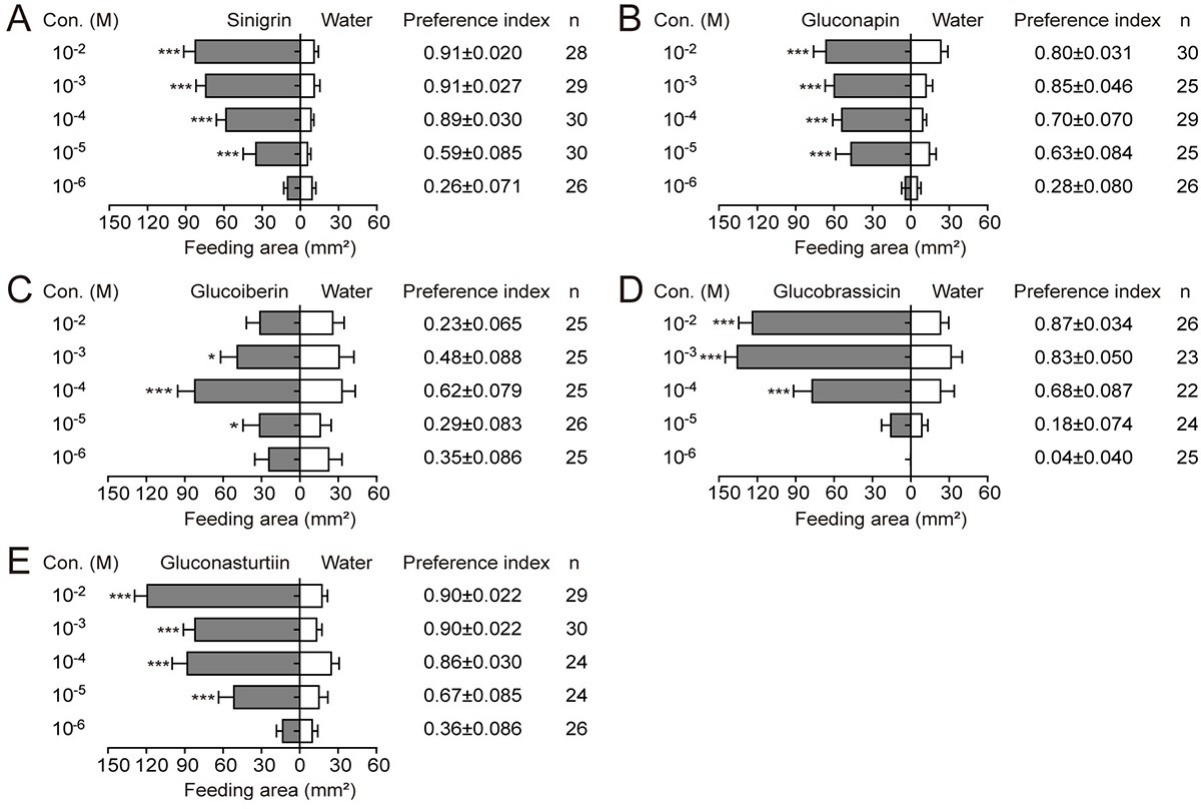
Feeding preference of the fifth instar larvae of *P*. *rapae* to glucosinolates. Cowpea leaf discs were used as the substrate for the two choice assays of fifth instar *P*. *rapae* larvae. The upper surface of each disc was treated with 20 μL of glucosinolate solutions. Each control disc was supplied with the same volume of water. The concentration gradients of (**A**) sinigrin, (**B**) gluconapin, (**C**) glucoiberin, (**D**) glucobrassicin, and (**E**) gluconasturtiin all ranged from 10^−6^ to 10^−2^ M. When the total feeding area was larger than 25% or after 24 h feeding, the area of each disc consumed by larvae was measured, and the feeding preference index was calculated. Differences in feeding amounts on treated and control discs were tested by paired Student’s *t*-test. *n* represents the replicates of larvae and are labeled in the figures. Data are presented as mean ± SEM. * *P* < 0.05, ** *P* < 0.01, *** *P* < 0.001.

The cabbage, *Brassica oleracea*, one of main host plants for *P*. *rapae*, contains five main glucosinolates. Sinigrin, gluconapin and glucoiberin are aliphatic glucosinolates, glucobrassicin and gluconasturtiin are indolic and aromatic glucosinolates, respectively [9,10]. We applied a series of concentrations of glucosinolates on leaf discs from the non-host plant cowpea (Fabaceae) and determined the feeding preference of *P*. *rapae* larvae to these glucosinolates in a dual choice test. Sinigrin, gluconapin, glucobrassicin and gluconasturtiin strongly stimulated larval feeding, and the feeding preference indexes (PIs) increased with increased concentration (Figs 1A, 1B, 1D, 1E, and S1). Glucoiberin also had a stimulating effect, especially at the concentration of 10^−4^ M (Figs 1C and S1). However, the PI did not correlate with glucoiberin concentrations (Fig 1C). The stimulatory activity of sinigrin, gluconapin, glucoiberin and gluconasturtiin to larval feeding started at the dose of 10^−5^ M (Fig 1A–1C, and 1E), whereas that of glucobrassicin started at 10^−4^ M (Fig 1D). At the concentration of 10^−4^ M, sinigrin was the most effective feeding stimulant (PI = 0.89 ± 0.030), followed by gluconasturtiin (0.86 ± 0.030), gluconapin (0.70 ± 0.070), glucobrassicin (0.68 ± 0.087), and glucoiberin (0.62 ± 0.079).

### The selectivity and sensitivity of taste sensilla of *P*. *rapae* larvae and adults to glucosinolates

The taste sensilla on both larval mouthparts and adult tarsi of *P*. *rapae* are sensitive to glucosinolates [17,19]. However, the sensilla responding to different glucosinolates have not been categorized. Thus, we systematically mapped the electrophysiological response profiles of these sensilla to glucosinolates.

**Fig 2:**
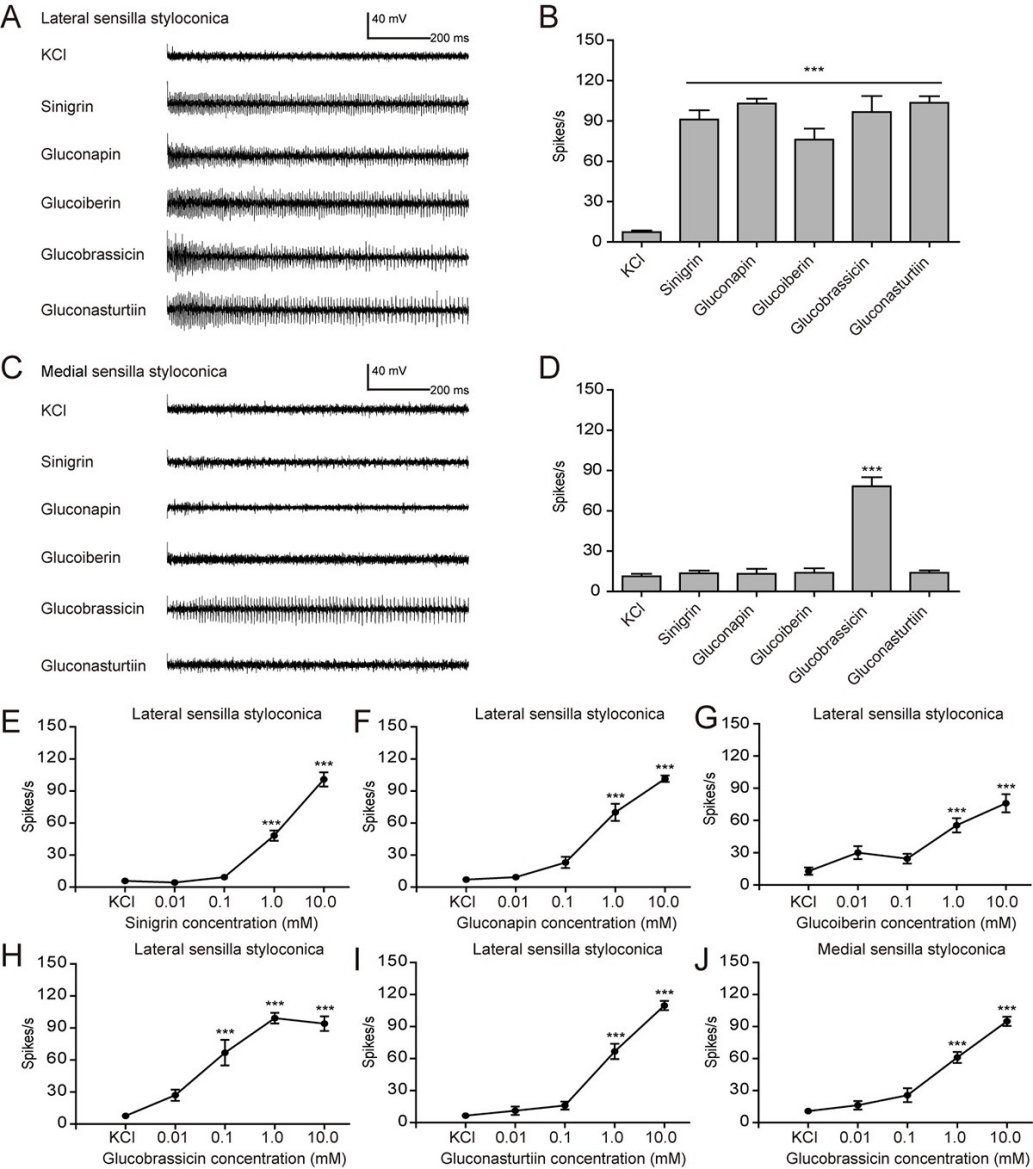
Response properties of sensilla styloconica on larval maxilla of *P*. *rapae* to glucosinolates. (**A**) Representative responses and (**B**) spike frequencies of lateral sensilla styloconica (*n* = 10); (**C**) representative responses and (**D**) spike frequencies of medial sensilla styloconica (*n* = 11). All tested glucosinolates were at 10 mM, and 2 mM KCl was used as control. (**E**-**I**) Dose-response curves of lateral sensilla styloconica to sinigrin (*n* = 10–11), gluconapin (*n* = 10–12), glucoiberin (*n* = 10), glucobrassicin (*n* = 5–7), and gluconasturtiin (*n* = 10), respectively; (**J**) dose-response curves of medial sensilla styloconica to glucobrassicin (*n* = 6–8). Data are presented as mean ± SEM. One-way ANOVA with Tukey HSD test was used. *** *P* < 0.001, compared with KCl control.

First, we determined the selectivity and sensitivity of two pairs of sensilla styloconica in the larval maxillary galea. We confirmed that a single neuron in the lateral sensilla styloconica responded with equal frequency to sinigrin, gluconapin, glucoiberin, glucobrassicin and gluconasturtiin at a concentration of 10 mM (Fig 2A and 2B). By contrast, one neuron in the medial sensilla styloconica specifically responded to glucobrassicin, whereas it did not respond to sinigrin, gluconapin, glucoiberin and gluconasturtiin (Fig 2C and 2D). The spike frequency of all these sensilla was positively correlated with the concentration of the respective glucosinolates (Figs 2E–2I and S2A–S2E). The lateral sensilla styloconica showed appreciable responses to as low as 1 mM of sinigrin, gluconapin, glucoiberin and gluconasturtiin (Figs 2E–2G and 2I, S2A–S2C, and S2E), while the responses of the lateral and medial sensilla styloconica for glucobrassicin respectively started from 0.1 mM and 1 mM (Figs 2H, 2J, S2D, and S2F), indicating that in larvae lateral sensilla styloconica were more sensitive to glucobrassicin than medial sensilla.

**Fig 3:**
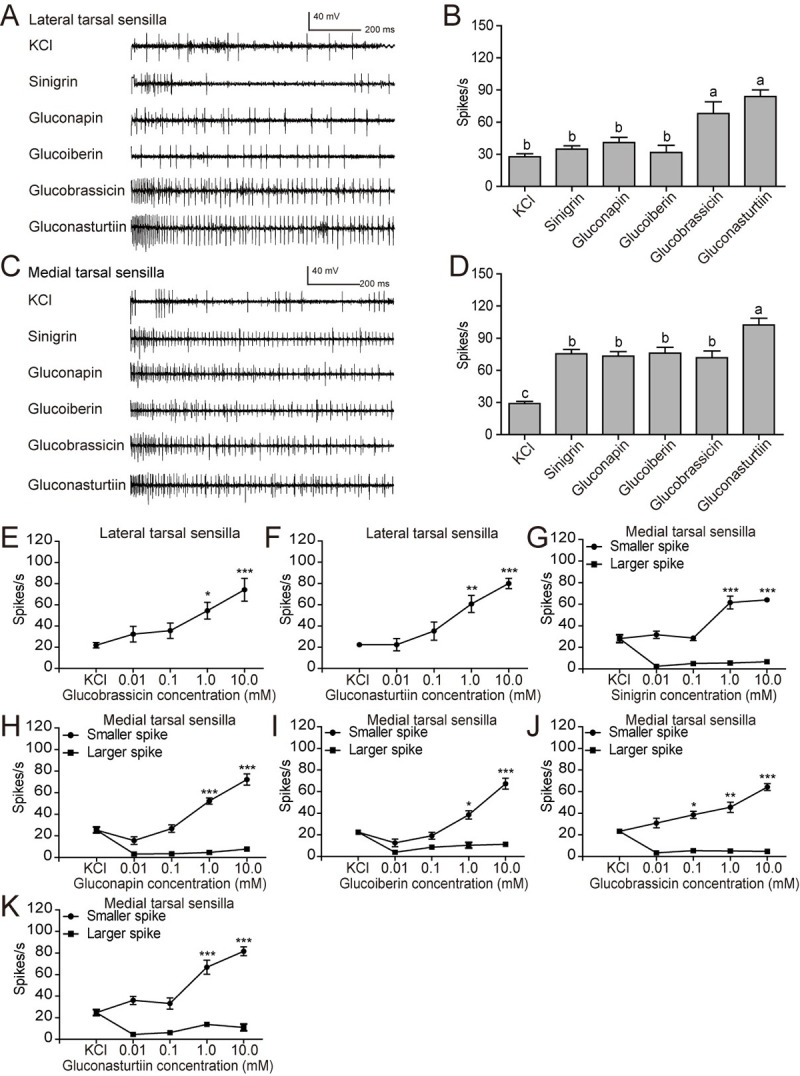
Response properties of taste sensilla on the fifth foreleg-tarsi of female *P*. *rapae* to glucosinolates. (**A**) Representative responses and (**B**) spike frequencies of female lateral tarsal sensilla (*n* = 7–15); (**C**) representative responses and (**D**) spike frequencies of female medial tarsal sensilla (*n* = 10–17). All tested glucosinolates were at 10 mM, and 2 mM KCl was used as control. (**E**, **F**) Dose-response curves from female lateral tarsal sensilla to gradient concentration of glucobrassicin (*n* = 6) and gluconasturtiin (*n* = 5); (**G**-**K**) dose-response curves of female medial tarsal sensilla to sinigrin (*n* = 10), gluconapin (*n* = 8), glucoiberin (*n* = 7–8), glucobrassicin (*n* = 8), and gluconasturtiin (*n* = 8), respectively. Data are presented as mean ± SEM. One-way ANOVA with Tukey HSD test was used. Different letters labeled indicate significant differences. * *P* < 0.05, ** *P* < 0.01, *** *P* < 0.001, compared with KCl control.

Second, we set out to determine the selectivity and sensitivity of two clusters of trichoid taste sensilla, lateral tarsal sensilla and medial tarsal sensilla, on female foreleg tarsi to glucosinolates. We found that the lateral tarsal sensilla were sensitive to glucobrassicin and gluconasturtiin at a concentration of 10 mM, but not to sinigrin, gluconapin and glucoiberin (Fig 3A and 3B). The dose-response curves showed that 1.0 mM of glucobrassicin and gluconasturtiin was sufficient to induce the responses of the lateral tarsal sensilla (Figs 3E, 3F, S3A and S3B). The medial tarsal sensilla responded to all the tested glucosinolates, with a relatively higher frequency to gluconasturtiin (Fig 3C and 3D). However, just as previously reported [19], two types of spikes were recorded in the medial tarsal sensilla when stimulated by glucosinolates, smaller amplitude spikes with a high frequency and larger amplitude spikes with a low frequency (Figs 3, S3, and S4A). The dose-response curves showed that only the frequencies of the smaller amplitude spikes increased with the glucosinolate concentrations (Figs 3G–3K and S3C–S3G). Threshold for activation of medial tarsal sensilla was seen at 0.1 mM for glucobrassicin, whereas the threshold occurred at a 10 times higher concentration for sinigrin, gluconapin, glucoiberin and gluconasturtiin (Figs 3G–3K and S3C–S3G).

**Fig 4:**
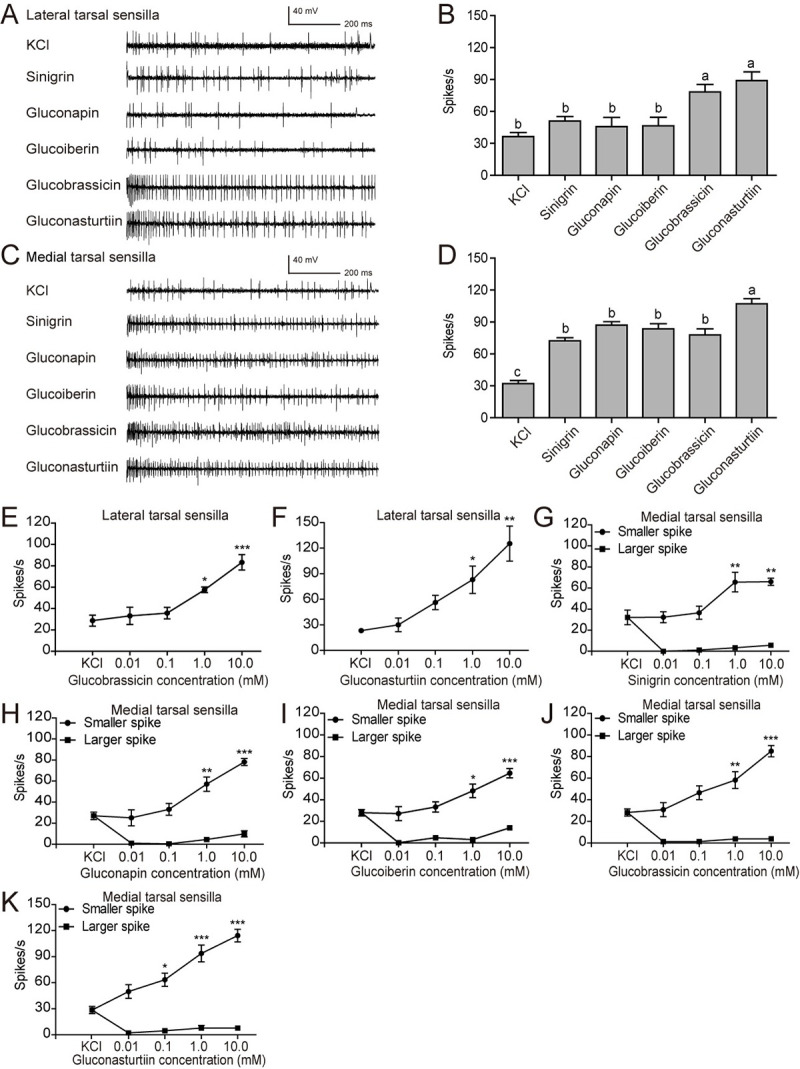
Response properties of taste sensilla on the fifth foreleg-tarsi of male *P*. *rapae* to glucosinolates. (**A**) Representative responses and (**B**) spike frequencies of lateral tarsal sensilla (*n* = 9–16); (**C**) representative responses and (**D**) spike frequencies of medial tarsal sensilla (*n* = 12–18). All tested glucosinolates were at 10 mM, and 2 mM KCl was used as control. (**E**, **F**) Dose-response curves of lateral tarsal sensilla to glucobrassicin (*n* = 6–7) and gluconasturtiin (*n* = 3). (**G**-**K**) Dose-response curves of medial tarsal sensilla to sinigrin (*n* = 6–7), gluconapin (*n* = 6–8), glucoiberin (*n* = 5–7), glucobrassicin (*n* = 7–8), and gluconasturtiin (*n* = 7), respectively. Data are presented as mean ± SEM. One-way ANOVA with Tukey HSD test was used. Different letters labeled indicate significant differences. * *P* < 0.05, ** *P* < 0.01, *** *P* < 0.001, compared with KCl control.

Finally, we tested the response profile of male tarsi to glucosinolates. We found a similar distribution pattern of the tarsal taste sensilla in the two sexes. The selectivity, spike type and sensitivity of taste sensilla to glucosinolates was also comparable between males and females. The lateral tarsal sensilla were sensitive to glucobrassicin and gluconasturtiin at a concentration of 10 mM (Fig 4A and 4B). The medial tarsal sensilla responded to all the tested glucosinolates, with a relatively higher frequency to gluconasturtiin (Fig 4C and 4D). Only the smaller amplitude spike frequency of tarsal medial sensilla in males was positively correlated with the concentration of glucosinolates (Figs 4G–4K, S4B, and S5). The concentration required for activating lateral tarsal sensilla by glucobrassicin and gluconasturtiin was 1.0 mM (Figs 4E, 4F, S5A and S5B). The activation threshold concentration for gluconasturtiin in medial tarsal sensilla was 0.1 mM, while a 10 time’s higher threshold was found for sinigrin, gluconapin, glucoiberin and glucobrassicin (Figs 4G–4K and S5C–S5G).

Based on the above results, the lateral sensilla styloconica of larvae and medial sensilla of adult tarsi responded to sinigrin and four other diagnostic glucosinolates in an indistinguishable manner, while the medial sensilla styloconica of larvae were exclusively tuned to glucobrassicin, and lateral sensilla of adult tarsi were only tuned to glucobrassicin and gluconasturtiin. Clearly, more than one GRN is tuned to glucosinolates in *P*. *rapae*.

### Expression patterns of gustatory receptors in taste organs of *P*. *rapae*

**Fig 5:**
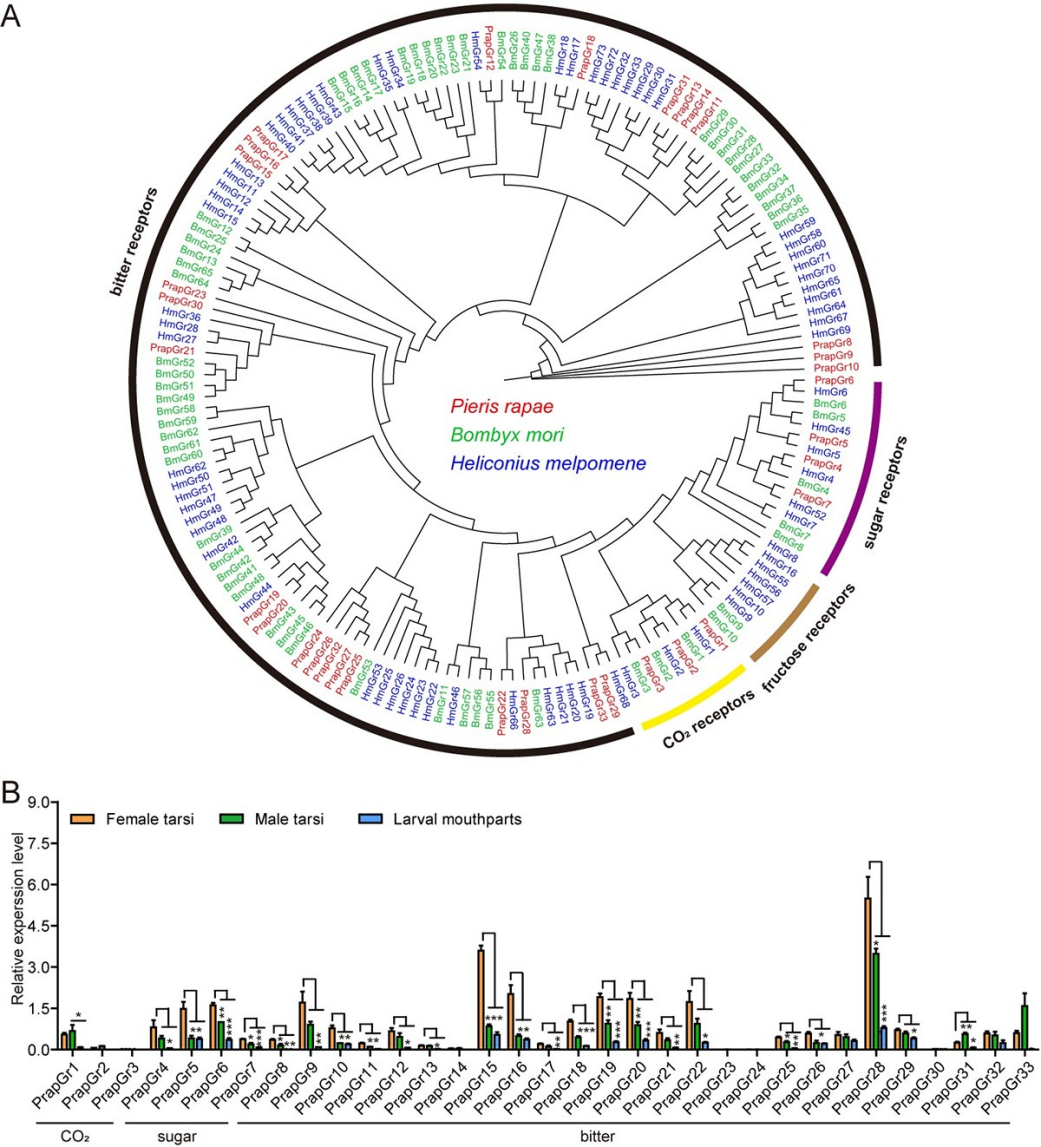
Phylogenetic relationships and tissue expression patterns of *GR* genes in *P*. *rapae*. (**A**) Phylogenetic tree of candidate GRs from *P*. *rapae* and other Lepidoptera species. Phylogenetic tree was constructed using Maximum likelihood phylogenies with JTT + F + G4 model. The purple, brown, yellow and black arcs represent sugar receptors, fructose receptors, CO_2_ receptors and bitter receptors, respectively. Prap, *Pieris rapae* (red); Bm, *Bombyx mori* (green); Hm, *Heliconius melpomene* (blue). (**B**) Expression profiles of candidate *GR* genes. Transcript levels were detected by qRT-PCR and calculated based on the 2^−ΔΔCt^ method. *n* = 3. Data are presented as mean ± SEM. One-way ANOVA with Tukey HSD test was used. * *P* < 0.05, ** *P* < 0.01, *** *P* < 0.001, compared with female tarsi.

Having demonstrated that GRNs housed in taste sensilla strongly respond to glucosinolates, we next explored the candidate GRs expressed in these taste neurons responding to glucosinolates. Based on transcriptome sequencing analyses, a repertoire of 33 putative *GRs* were identified (S1 Table). Phylogenetic analysis of GRs from *B*. *mori* [43] and *Heliconius melpomene* [44] suggested that PrapGr1-3 were CO_2_, and PrapGr4-7 and PrapGr8-33 belonged to sugar and bitter receptor subfamilies, respectively (Fig 5A). Since glucosinolates are plant allelochemicals, they are presumably detected by bitter receptors.

We first found that the variety and expression of *GRs* in the adult tarsi were generally higher than those in larval mouthparts revealed by TPM (transcripts per kilobase of exon model per million mapped reads) values of candidate *GRs*, which is consistent with the higher number of glucosinolate-responsive taste sensilla on adult tarsi than on larval mouthparts.

Based on TPM value of *GRs* in adult tarsi, the most abundant bitter receptor was *PrapGr22*, followed by *PrapGr33* and *PrapGr28*, and then *PrapGr15*, *PrapGr10* and *PrapGr8* (S6 Fig). In the larval mouthparts, the bitter receptor with the highest TPM value was *PrapGr22*, followed by *PrapGr28*, and the TPM value of other bitter receptors were much lower (S6 Fig). Next, we used qRT-PCR to verify the expression patterns of the aforementioned *GRs*. In contrast to the TPM value resulting from the transcriptomic dataset, the most abundantly expressed *GR* in female tarsi was *PrapGr28*, the second was *PrapGr15*, while *PrapGr22* was third per PCR quantification (Fig 5B). The expression pattern of bitter receptors in male tarsi was similar to that in female tarsi with *PrapGr28* being the highest, but the expression level of most bitter receptors was lower than that in female tarsi (Fig 5B). The expression levels of the three genes in larval mouthparts were much lower than in adult tarsi, but had a similar expression ranking (Fig 5B). In light of the consistence between the predominant expression of *PrapGr28* and *PrapGr15* in adult tarsi and larval mouthparts and the prominent glucosinolate sensitivity found in these taste sensilla tissues, we speculated that the bitter receptors *PrapGr28* and *PrapGr15* might be involved in the chemoreception of glucosinolates. These two receptors were predicted to have typical GR characteristics with seven transmembrane domains, and shared 14% identity at the amino acid level (S7A and S7B Fig). In addition, the identified bitter receptors in *P*. *rapae* showed low sequence identities based on our transcriptomic dataset (S7C Fig and S1 Table).

### GR functional analysis with the *Xenopus* oocyte expressing system

**Fig 6:**
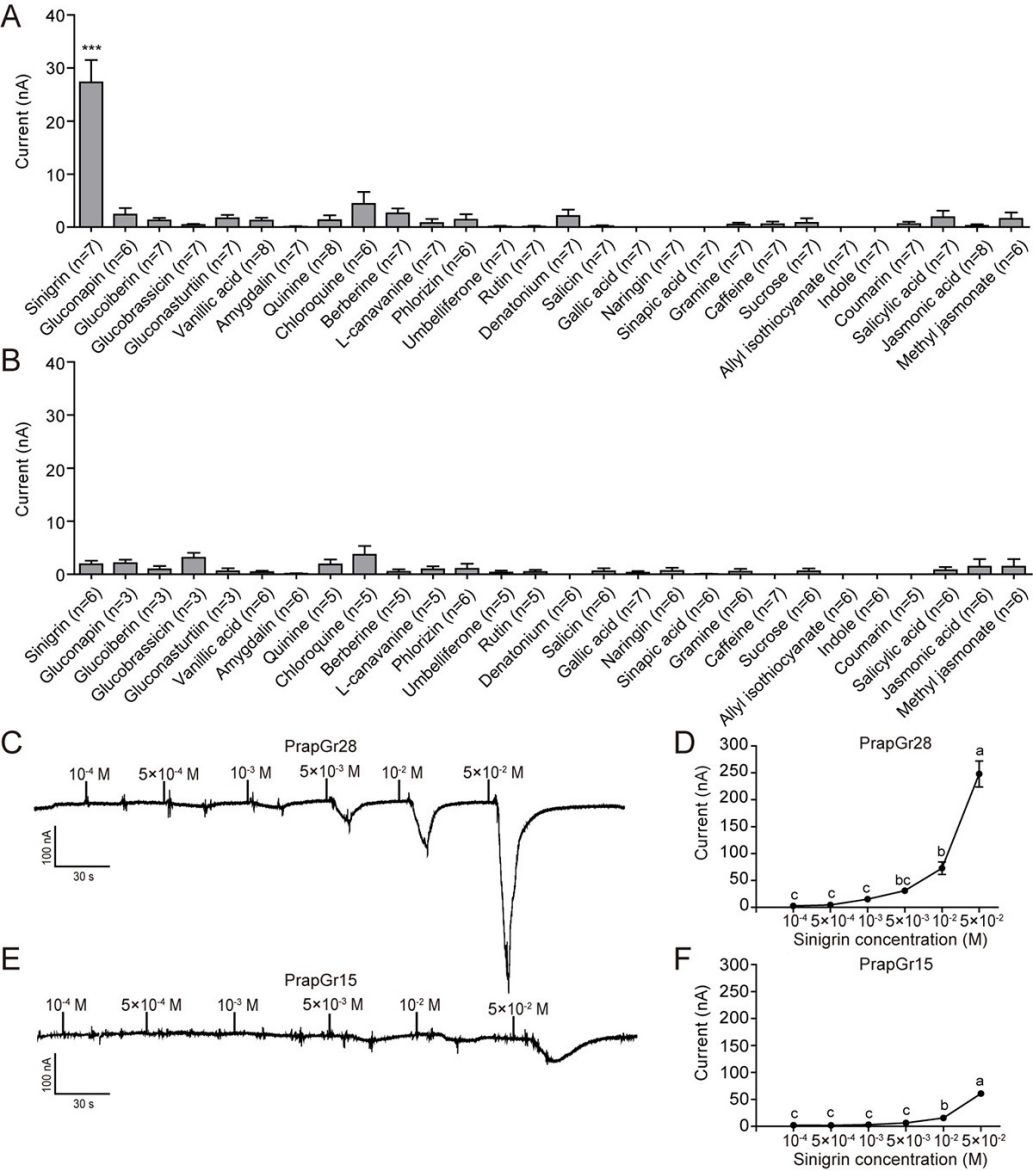
Functional analysis of PrapGrs in *Xenopus* oocytes. (**A, B**) Response profiles of *Xenopus* oocytes expressing *PrapGr28* (**A**) and *PrapGr15* (**B**) in response to compounds at 1 mM. *** *P* < 0.001. *n* represents the number of oocytes and are labeled in the figures. (**C**-**F**) Inward current responses (**C**, **E**) and dose-response curve (**D**, **F**) of *Xenopus* oocytes expressing *PrapGr28* (*n* = 5–6), and *PrapGr15* (*n* = 5) stimulated with a range of sinigrin concentrations, respectively. Data are presented as mean ± SEM. Different letters labeled indicate significant differences. One-way ANOVA with Tukey HSD test was used.

To functionally characterize PrapGr28 and PrapGr15, we expressed them individually or in a combination in *Xenopus* oocytes, and then recorded the response to a panel of chemical stimuli including five glucosinolates by two-electrode voltage-clamp recordings. We found that the oocytes expressing *PrapGr28* selectively responded to sinigrin, while the mock oocytes did not respond to 1 mM sinigrin (Figs 6A, S8A, and S10). The oocytes expressing *PrapGr28* also showed dose-dependent responses to sinigrin, and a dosage of 5 mM induced a strong response current (Fig 6C and 6D). The oocytes expressing *PrapGr15* did not show an obvious response to any tested stimuli at 1 mM (Figs 6B, S8B, and S10), and did not exhibit dose-dependent response until at a concentration of 50 mM sinigrin it produced a small response (Fig 6E and 6F). *PrapGr28* and *PrapGr15* both expressed in oocytes did not respond significantly to any stimulus at 1 mM (S9A, S9B, and S10 Figs). However, these oocytes showed a clear dose-response curve to sinigrin although it was much lower than that of the oocytes expressing single *PrapGr28* (S9C and S9D Fig). We conclude that the presence of PrapGr28 causes the response to sinigrin in oocytes expressing both *PrapGr15* and *PrapGr28*.

### GR functional analysis in *Drosophila* expressing system

**Fig 7:**
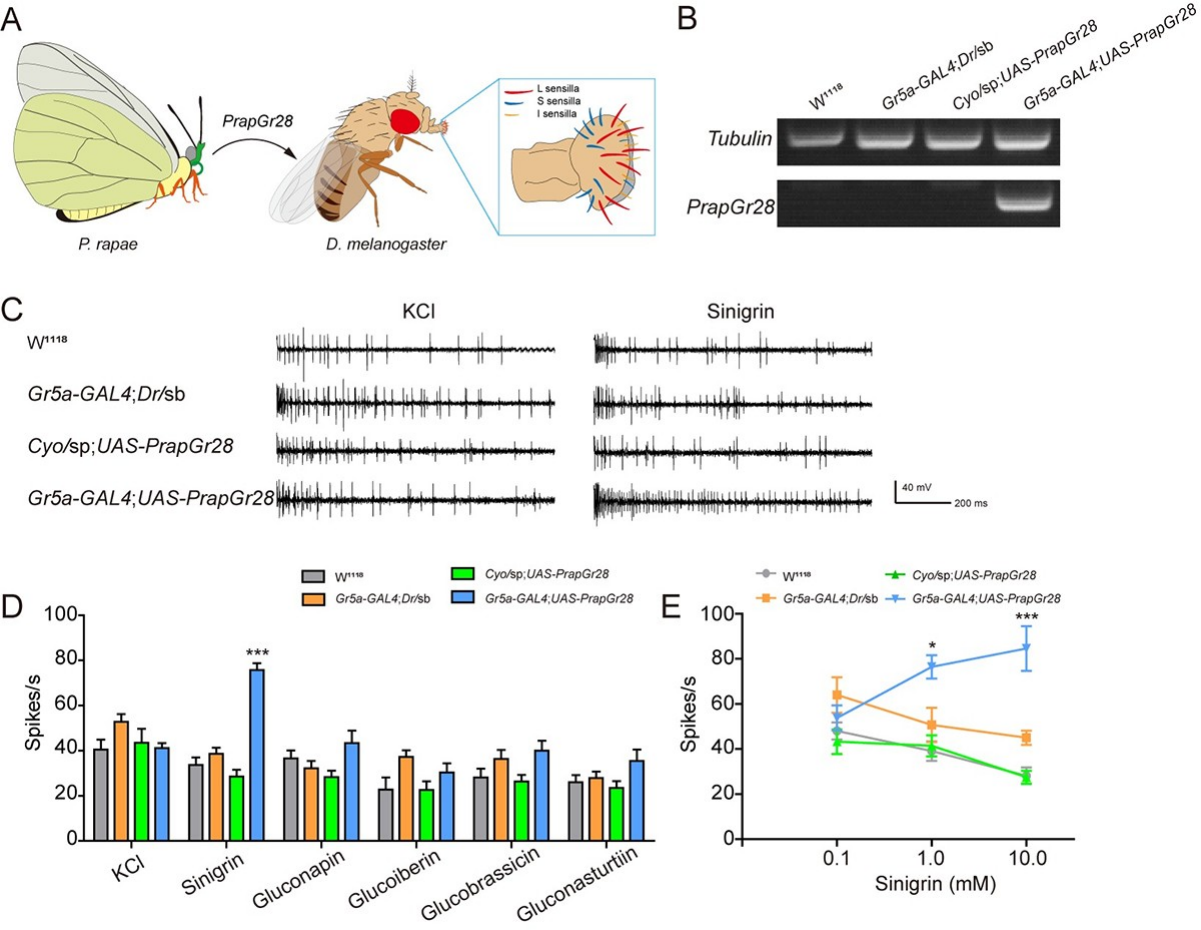
The presence of *PrapGr28* confer sinigrin sensitivity to *D*. *melanogaster* sweet neuron. (**A**) Schematic diagram of *PrapGr28* expressed in sweet neuron of *D*. *melanogaster* L-type sensilla. (**B**) Expression of *PrapGr28* in the labellum of fly lines. *Tubulin* was used as reference gene. (**C**) Representative responses and (**D**) spike frequencies of L-type sensilla on the labellum of fly lines to glucosinolates. KCl, *n* = 10–11; sinigrin, *n* = 10–11; gluconapin, *n* = 8–11; glucoiberin, *n* = 9–11; glucobrassicin, *n* = 8–11; gluconasturtiin, *n* = 9–11. All tested glucosinolates were at 10 mM, and 1 mM KCl was used as control. (**E**) Dose-response curves of L-type sensilla to sinigrin. *n* = 7–12. Data are presented as mean ± SEM. One-way ANOVA with Tukey HSD test was used. * *P* < 0.05, *** *P* < 0.001, compared with control flies.

To further confirm the function of PrapGr28, we ectopically expressed *PrapGr28* into *Drosophila* sweet GRNs by *Gr5a-GAL4* (Fig 7A) that are normally electrophysiologically silent to bitter compounds [45]. The Gr5a GRNs are mainly distributed in the large (L-type) sensilla across the entire labial palp in *D*. *melanogaster* [46,47], which makes these GRNs expressing *PrapGr28* more accessible to tip recording. First, we confirmed *PrapGr28* was expressed in the *Drosophila* labellum by reverse transcription-PCR (RT-PCR) (Fig 7B). Second, to ensure the tested sensilla, we first stimulated L-type sensilla with 10 mM sucrose. If they responded to sucrose (S11 Fig), we proceeded to test the sensitivity of these sensilla to the glucosinolate compounds. The L-type sensilla from parental lines (*UAS*-*PrapGr28* and *Gr5a-GAL4* flies) did not respond to sinigrin, whereas only the L-type sensilla from the *Gr5a*-*GAL4*;*UAS*-*PrapGr28* fly line specifically responded to sinigrin (Figs 7C, 7D, and S12), which resembled the response profile of PrapGr28 expressed in oocytes. We also found that the *Drosophila* L-type sensilla expressing *PrapGr28* responded to sinigrin in a dose-dependent manner (Figs 7E and S13). In addition, we tested whether the flies expressing *PrapGr28* behaviorally prefer the food laced with sinigrin. Although the wild type flies avoided the food containing 10 mM of sinigrin, the presence of PrapGr28 in the sweet neurons reduced the aversive effects of sinigrin to flies (S14 Fig).

### Location of *GRs* expression in the foreleg tarsi of adults

We also tested whether *PrapGr28* and *PrapGr15* were co-expressed in the GRNs in adult tarsi. By *in situ* hybridization, we observed *PrapGr28* expressing cells in female and male foreleg tarsi of *P*. *rapae*, but failed to detect the cells expressing *PrapGr15*, which is presumably due to the lower expression level of *PrapGr15* in tarsi (S15 Fig).

### Effect of knockdown of *PrapGr28* on the sensitivity of taste sensilla in adults to glucosinolates

**Fig 8:**
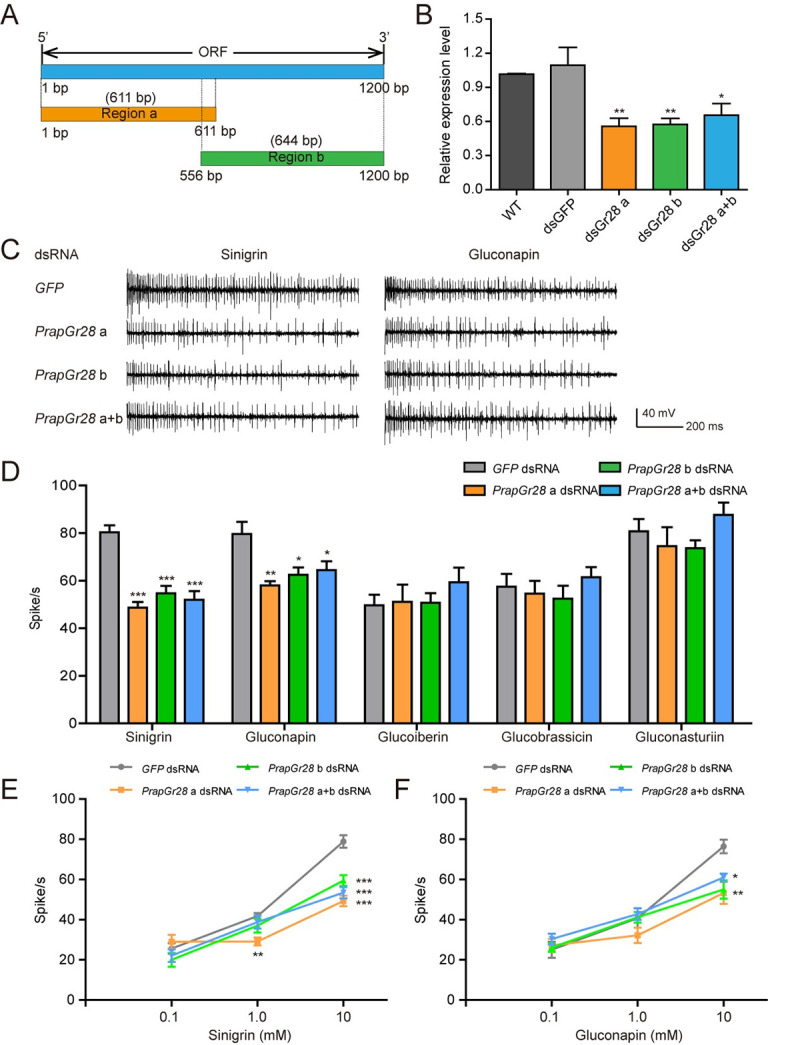
RNA interference of *PrapGr28* suppresses the response to glucosinolates in female butterflies. (**A**) Schematic of *PrapGr28* and the regions used for dsRNA synthesis. Region a and b, named *PrapGr28* a and *PrapGr28* b, respectively, are portions of the coding region of *PrapGr28* used for preparation of dsRNA. (**B**) Relative expression levels of *PrapGr28* in dsRNA-injected adult butterflies. *n* = 4–5. WT, wild type without injection; dsGFP, *GFP* dsRNA; dsGr28 a, *PrapGr28* a dsRNA; dsGr28 b, *PrapGr28* b dsRNA; dsGr28 a+b, *PrapGr28* a+b dsRNA. (**C**) Representative responses and (**D**) smaller amplitude spike frequencies elicited by glucosinolates in the medial sensilla of the fifth prothoracic tarsi. Sinigrin, *n* = 10–13; gluconapin, *n* = 9–14; glucoiberin, *n* = 7–13; glucobrassicin, *n* = 6–12; gluconasturtiin, *n* = 6–14. All tested glucosinolates were at 10 mM. (**E**, **F**) Dose-response curves from medial sensilla on the prothoracic tarsi of female butterflies to gradient concentration of sinigrin (*n* = 5–9) and gluconapin (*n* = 5–9), respectively. Data are presented as mean ± SEM. One-way ANOVA with Tukey HSD test was used. * *P* < 0.05, ** *P* < 0.01, *** *P* < 0.001, compared with the control.

To further validate the function of PrapGr28 *in vivo*, we injected *PrapGr28* dsRNA into female pupae and verified the knockdown of *PrapGr28* in the female tarsi and the sensitivity of taste sensilla to glucosinolates after eclosion. To test the effectiveness of knockdown of full-length *PrapGr28*, we set up three dsRNA treated groups, *PrapGr28* a dsRNA, *PrapGr28* b dsRNA, and *PrapGr28* a+b dsRNA (a mixture of *PrapGr28* a and b dsRNA with same volume). *PrapGr28* a and *PrapGr28* b target the different regions of the gene *PrapGr28* though partially overlapping (Fig 8A). The expression of *PrapGr28* in three groups of *PrapGr28* dsRNA decreased by 45.1%, 43.5% and 35.53% by comparing with the wild type butterflies, and 49.0%, 47.8% and 40.2% by comparing with the *GFP* dsRNA group (Fig 8B). To evaluate the RNAi effect of *PrapGr28* on electrophysiological responses of female tarsi to glucosinolates, we also analyzed the frequencies of the smaller amplitude spikes from medial tarsal sensilla. In the three *PrapGr28* dsRNA groups, the smaller spike frequencies recorded from medial tarsal sensilla in response to 10 mM of sinigrin and gluconapin were reduced, but the sensitivity to glucoiberin, glucobrassicin and gluconasturtiin were not affected (Figs 8C, 8D, and S16). Through detecting the response of medial tarsal sensilla to sinigrin and gluconapin at different concentrations, we found that only the sensitivity of taste sensilla of individuals from the *PrapGr28* a dsRNA group was significantly reduced among three *PrapGr28* dsRNA groups when stimulated by 1 mM sinigrin (Figs 8E and S17A). However, the sensitivity to gluconapin in RNAi butterflies was only markedly reduced at a higher concentration (10 mM) (Figs 8F and S17B). In addition, the knockdown of *PrapGr28* had no effect on the spike frequency of the lateral tarsal sensilla of adults stimulated by glucobrassicin and gluconasturtiin (S18 Fig).

## Discussion

Glucosinolates, as a group of important bioactive compounds found mainly in cruciferous plants, are long known to act as token stimuli to *P*. *rapae* and other crucifer specialist insects, but the molecular mechanism by which such compounds are sensed has been a mystery. In this study, we tested the behavioral and electrophysiological responses to glucosinolates of this butterfly, and combined with evidence from transcriptome analyses of taste organs and functional characterization of GRs, this work revealed that *PrapGr28* codes for a receptor in *P*. *rapae* tuned to sinigrin, one of the most common and abundant glucosinolates in cruciferous plants.

### Glucosinolates as token stimuli for *P*. *rapae* to recognize Cruciferae plants

In 1910, Verschaffelt first reported that glucosinolates can serve as token stimuli for larvae of *P*. *brassicae* by demonstrating stimulation of feeding from a non-host plant [48]. Half a century later, it was discovered that glucosinolate-sensitive-GRNs were contained in two sensilla styloconica on the maxillary galea and mediate the feeding preference of *P*. *brassicae* larvae for glucosinolates [28]. One GRN in the lateral sensillum styloconicum responded to six structurally different glucosinolates, and another GRN in the medial sensillum styloconicum was only tuned to aromatic glucosinolates, however, indolic glucosinolates have not been tested [28]. Later on, sinigrin and gluconasturtiin were reported as feeding stimulants for *P*. *rapae* larvae, and two GRNs in the lateral sensillum styloconicum were activated by gluconasturtiin [16,17]. Adult females of *P*. *brassicae*, *P*. *napi oleracea* and *P*. *rapae* also use glucosinolates as oviposition cues [15,18,49]. The butterflies explore the surface of plants using contact chemosensory hairs on their tarsi [50]. Tip recordings from the medial tarsal sensilla in *P*. *napi oleracea* and *P*. *rapae* females have showed that two neurons in these sensilla, characterized by spikes of differing amplitude, are sensitive to a number of different glucosinolates. The one characterized by smaller amplitude spikes respond specifically to some glucosinolates, while the other one, with larger amplitude spikes, most likely is a deterrent neuron responding to cardenolides [19,24]. In this study, we only found dose dependent responses of the GRN from which the smaller amplitude spike was recorded. These token stimulus neurons are thought to act as ‘labelled lines’, along which specific information is transferred to the brain and the electrophysiological activity of which correlates quantitatively with the strength of the behavioral response [3,26]. Neural activity of these ‘labelled line’ signals allows adults and larvae to discriminate cruciferous plant species from plants lacking glucosinolates.

The adaptation of several *Pieris* species to host plants can be partly achieved through the differential tasting of glucosinolates. Glucoiberin is a weaker stimulant of feeding and oviposition of *P*. *rapae* [19,51], but can strongly stimulate the oviposition of *P*. *napi oleracea* [51]. It is mainly found in non-cultivated Cruciferae, such as candytuft *Iberis amara* and wormseed mustard *Erysimum cheiranthoides*, which are major host plants of *P*. *napi oleracea*, but not of *P*. *rapae* [51,52]. In addition to glucosinolates, these plants also biosynthesize cucurbitacins or cardenolides, which are strong oviposition deterrents for *P*. *rapae*, but not for *P*. *napi oleracea* [49,51]. It appears that not all glucosinolates have the same activity for any one insect species, nor is the activity of one compound equal across specialist species [4].

In this study, we validate that the lateral sensilla styloconica of *P*. *rapae* larvae promiscuously respond to all five tested glucosinolates, while the medial sensilla styloconica only respond to glucobrassicin. These results are similar to the previously reported in *P*. *brassicae* larvae albeit the glucosinolates tested were not exactly the same [28]. A difference is that in *P*. *rapae*, the indolic glucosinolate glucobrassicin activates both lateral and medial sensilla styloconica of larvae, whereas in *P*. *brassicae* this dual activation was found for the aromatic glucosinolates glucotropaeolin and gluconasturtiin, however, glucobrassicin has not been tested [28].

In *P*. *rapae* adults, we confirm that the medial tarsal sensilla, like the lateral sensilla styloconica on the larval maxilla, respond to all tested glucosinolates, which is in accordance with a previous study [19]. The lateral tarsal sensilla of *P*. *rapae* adults contain one receptor neuron sensitive to glucobrassicin and gluconasturtiin, whereas the medial sensilla styloconica on the larval maxilla is only sensitive to glucobrassicin, suggesting that GRs of the adult and larval neurons overlap but are not identical.

The existence of two types of GRNs in both larvae and adults with distinct response spectra to glucosinolates suggests subtle phytochemical sensing mechanisms in host-plant selection. It is plausible that the balance of inputs from the two types of specialist GRN allows the sensing of total glucosinolate concentration by the broadly tuned neuron, and the concentration of indolic and aromatic glucosinolates by the narrowly tuned neuron, in determining their feeding preference. If this hypothesis is true, it means that a combinatorial coding for glucosinolates operates in these species.

A combinatorial coding mechanism is likely to be adaptive since glucosinolates differ in toxicity, aliphatic compounds being the most toxic class [53–55]. Enzymatic hydrolysis in the larval gut of aliphatic glucosinolates by the thioglucosidase enzyme myrosinase present in cruciferous plants results in highly toxic isothiocyanates. As a biochemical adaptation to cope with isothiocyanate toxicity, *P*. *rapae* and other pierid caterpillars have a unique protein in their gut that has been coined nitrile-specifier protein that diverts the hydrolytic cleavage and molecular rearrangement of aliphatic aglycones to produce less toxic nitriles that are then excreted [53]. Enzymatic hydrolysis of the aromatic glucosinolates gives rise to formation of a strongly toxic cyanide that is detoxified by β-cyanoalanine synthases recently discovered in *P*. *rapae* [56]. Indolic glucosinolates and their breakdown products seem to exert low toxicity to *P*. *rapae*. Two lines of evidence support this notion: (1) the indolic glucosinolate glucobrassicin is the strongest oviposition stimulus for *P*. *rapae* among glucosinolates tested thus far [18,19]; (2) indolic glucosinolates commonly reach higher concentrations upon induction by *P*. *rapae* feeding than aliphatic compounds [57,58]. Differential toxicity of breakdown products in concert with feeding-induced changes in foliar glucosinolate profiles may have been selected for a discrimination mechanism that allows sensing of the ratio between total and specific glucosinolates, in particular indolic and aromatic glucosinolates, in both larvae and adults. This hypothesis is supported by the presence of two types of GRNs with different glucosinolate response profiles in the larval mouthparts and adult forelegs of *P*. *rapae*. These GRNs play an indispensable role in glucosinolate addiction of *Pieris* butterflies [19,24,28].

### Consistency between expression level and transcriptome analysis of gustatory receptor genes

Currently, GRs are mainly identified through transcriptome and genome sequencing, and candidate genes can be selected by sequence alignment and expression analysis. Because glucosinolates are non-volatile plant secondary substances, we surmised that their receptors could belong to bitter receptors. The foreleg tarsi are a major taste organ, and are equipped with plenty of taste sensilla [14,19]. The electrophysiological tip recordings indicate that two clusters of trichoid taste sensilla on the fifth tarsal segment of adults are sensitive to glucosinolates. Therefore, we reasoned that the bitter receptors highly expressed in tarsi are candidate glucosinolate receptors.

TPM or FPKM (fragments per kilobase of transcript per million fragments mapped) values calculated from RNA-sequencing (RNA-seq) data are commonly used to evaluate the expression level of candidate genes, but a further validation is warranted using qRT-PCR to verify the expression of important genes [59]. RNA-seq is a large-scale gene screening, reflecting the overall trend of gene expression change in whole samples, while qRT-PCR reflects individual gene expression relative to a control gene. When genes are sparsely expressed or duplicated, the quantification based on TPM or FPKM values are likely inconsistent with qRT-PCR results [59]. Due to the low expression levels of most bitter receptors [39,60], qRT-PCR verification of target gene expression is indispensable. In this study, the correlation of TPM values and qRT-PCR results of *P*. *rapae* GRs confirm this point. We finally determined that *PrapGr28* and *PrapGr15* are highly expressed in the tarsi as the functional target genes.

### Functional analysis of bitter receptors using a combination of heterologous and *in vivo* methods

Numerous bitter receptors have been sequenced in herbivorous insects, but only a few receptors have been functionally characterized. In recent years, several heterologous expression systems, such as the *Spodoptera frugiperda* 9 (Sf9) cell, Human Embryonic Kidney (HEK) 293 cell, *Xenopus* oocyte and *Drosophila* empty neuron systems, are commonly used in the functional analysis of chemosensory receptors in non-model insects [39,46,61]. Using Sf9 cells and RNAi methods, PxutGr1 has been shown to respond to synephrine, an oviposition stimulus for *P*. *xuthus* [39]. In addition, a complex formed by two CO_2_ receptors has been shown to be necessary for CO_2_ detection in cotton bollworms and mosquitoes in oocytes and clustered regularly interspaced short palindromic repeats (CRISPR)/CRISPR-associated protein-9 nuclease (Cas9) systems [62–64].

In this study, we first used the *Xenopus* oocyte expression system for functional analysis of candidate GRs, and then validated the GR functions via the *Drosophila* sugar GRNs and finally utilized the RNAi method to verify it. This is a successful practice of functional identification of GRs for insects through a combination of heterologous and *in vivo* methods. The *Xenopus* oocytes and *Drosophila* labellar sensilla expressing *PrapGr28* only responded strongly to sinigrin, confirming that PrapGr28 is a GR tuned to sinigrin in *P*. *rapae*. The expression of *PrapGr28* in *Drosophila* flies counteracted the aversion response to sinigrin which is mediated by the sinigrin sensitivity of bitter GRNs housed in taste sensilla in forelegs [65]. We also noticed that when expressed in the oocyte and *Drosophila* sugar neurons, *PrapGr28* expression resulted in responses to sinigrin but not to gluconapin, whereas knockdown of *PrapGr28* reduced the sensitivity of taste sensilla to both compounds. This difference may be due to the interaction between the bitter receptors. It is conceivable that PrapGr28 can form a dimer, or multimer with other receptors in response to gluconapin in *P*. *rapae*. In *Drosophila*, the responses to different bitter compounds rely on different co-expressed bitter receptors within an individual neuron type; for an individual bitter compound, the response also relies on different bitter receptors in different neuron types. For example, in S-b sensilla, the response to caffeine depended on Gr33a, Gr39a.a, Gr66a and Gr93a, while the response to azadirachtin depended on Gr33a and Gr66a; that to sparteine depended on Gr32a in S-a sensilla, but not in S-b sensilla [33]. Therefore, interpreting the results obtained by heterologous expression systems must be done with caution because there are still many unknowns when compared to the *in vivo* system. Moreover, inconsistent results may also be obtained using different systems. For example, *Eriocrania semipurpurella odorant receptor 4* (*EsemOR4*) expressed in HEK cells does not result in a response, to (*R*,*Z*)-6-nonen-2-ol and (*S*,*Z*)-6-nonen-2-ol whereas a response was observed when expressed in *Xenopus* oocytes [61]. Finding a consistent trend across multiple expression systems provides strong evidence for our conclusions, increasing the likelihood of deorphanizing a given chemosensory receptor.

### Key role of bitter receptors in taste perception of insects

Insects use bitter receptors to recognize bitter substances, and the number of bitter receptors is correlated with their host ranges. Polyphagous insects have a distinct expansion of bitter GRs compared with monophagous and oligophagous insects. For example, the oligophagous species *Manduca sexta* and *P*. *xylostella* contain 35 and 55 bitter receptors, respectively [36,37], while two highly polyphagous moths, *Spodoptera litura* and *S*. *frugiperda*, have more than 200 bitter GRs [38]. Thus, the possession of a large array of bitter receptors is a strategic adaptation to a wider host range in insects.

In general, bitter chemicals are aversive substances to insects. Studies of bitter coding in *Drosophila* revealed more complex and dynamic coding patterns: one compound activates multiple neurons; one neuron also responds to many bitter chemicals [32,33]. For example, escin activates both I-a and I-b sensilla on the *Drosophila* labellum and each sensillum also responds to other bitter chemicals [33]. In *Drosophila*, different ‘bitter’ GRNs co-expressed distinct subsets of bitter GRs, which are used to detect a rich variety of bitter substances [66]. The stereotype responses of GRNs in different sensilla can be shifted through expression or deletion of GRs [32,33,46]. In brief, the fruit fly uses combinatorial coding to perceive multiple bitter chemicals.

Although just a few bitter receptors have been deorphanized in Lepidoptera, it is highly plausible that ‘generalist’ deterrent GRNs also express a series of bitter GRs in lepidopteran larvae and adults, just like in *Drosophila*. In *B*. *mori*, both BmGr16 and BmGr18 respond to coumarin and caffeine, and BmGr53 is more broadly tuned to coumarin, caffeine and pilocarpine, which act as feeding deterrents [40]. However, we document here that GRs that detect typical ‘bitter’ compounds are expressed in token stimulus GRNs. This is particularly important because bitter compounds such as glucosinolates trigger appetitive behavior in specialist insects. In *P*. *xuthus*, a bitter receptor, PxutGr1, specifically confers a response to synephrine for host plant recognition for oviposition [39]. In this study, we not only injected the bitter receptors individually, but also co-injected both GRs in *Xenopus* oocytes, however, co-expression of *PrapGr28* and *PrapGr15* reduced the response intensity of the oocytes to sinigrin. These results show that a single receptor PrapGr28 is sufficient to respond to sinigrin in *P*. *rapae*, suggesting that it can function as a single receptor protein, as a homodimer or homomultimer. We cannot, however, rule out the possibility that other bitter receptors are also involved in sinigrin detection. The larval lateral styloconic GRN and the adult medial tarsal GRN likely co-expresses at least one other, possibly several GRs in addition to PrapGr28. Follow-up research is needed to establish how many GRs recognize glucosinolates in this species. The evidence strongly suggests nonetheless that PrapGr28 is involved in the chemosensory basis of host-plant specialization of *P*. *rapae*.

It is plausible that bitter receptors for detecting deterrents evolved to detect token stimuli, following the evolution of mechanisms to detoxify glucosinolates. The origin of token stimulus GRNs in specialist insects probably goes back to the ‘generalist’ deterrent GRNs through heritable changes in the processing of chemosensory cues in the peripheral sensilla or in the central nervous system (CNS). It would be very interesting to determine whether this is achieved by ‘bitter’ GRs being expressed in sugar neurons or through changes in how the CNS processes activity of specialized bitter GRNs [67]. The taste inputs from the mouthparts of *Pieris* larvae can be roughly divided into two categories. One is the input to stimulate feeding, which derives from sucrose, amino acid and glucosinolate (sinigrin) GRNs; the other is the input that inhibits feeding, which comes from deterrent GRNs [68]. How taste inputs are processed in an insect brain is still poorly understood. Calcium-imaging studies in *Drosophila* showed that deterrent neurons that drive aversive behavior and sweet/sugar neurons that drive appetitive behavior are processed by separate pathways in the brain [69]. However, electrophysiological studies in the moths *M*. *sexta* and *Heliothis virescens* showed that the second-order neurons from the subesophageal zone respond to diverse taste stimuli, including neurons that were activated by some deterrents as well as sucrose [30,70,71]. To understand how signals from glucosinolate GRNs are processed in the brain of *P*. *rapae*, it is necessary to analyze the anatomical, functional, and behavioral characteristics of the second-order taste neurons concerned.

In summary, we reveal that PrapGr28 is a GR tuned to sinigrin, a potent stimulant in larval feeding and adult oviposition by *P*. *rapae*, thereby providing a comprehensive approach to functional analyses of bitter GRs in herbivorous insects. However, which GRs are responsible for sensing other glucosinolates is still unknown, and needs to be determined in subsequent experiments. CRISPR-Cas9 genome editing provides a good opportunity to tackle this issue because it would directly and fundamentally illuminate the involvement of the target receptors. The identification of genes coding for glucosinolate GRs in crucifer specialist insects not only contributes to revealing the chemosensory basis of host-plant specialization in insects, but also has important significance for the comprehensive understanding of insect-plant co-evolution.

## Methods

### Ethics statement

All the experimental protocols of animal experimentation were approved by the Animal Care and Use Committee of Institute of Zoology, Chinese Academy of Sciences (Protocol Number IOZ17090-A).

### Plant culture and animal rearing

#### Plant culture

Seeds of cabbage *Brassica oleracea* (Zhong Gan No.15 (F1)) and cowpea *Vigna sinensis* (Cui Jiang) were purchased from the Institute of Vegetables and Flowers, Chinese Academy of Agricultural Sciences. The seeds of cabbage were sown in garden soil, and two week-seedlings were raised in polypots and grown in a climate chamber at *ca*. 25°C, with a 16 hr light: 8 hr dark cycle. Six to eight weeks old cabbage plants were used for rearing the larvae of *P*. *rapae*. The seeds of cowpea were sown in polypots, and grown in a climate chamber under the above conditions. Two to three weeks old cowpea plants were used as a substrate for larval bioassays of *P*. *rapae*.

#### Insect rearing

*Pieris rapae* were collected from a cabbage field in Luoyang, Henan Province, China. The larvae were reared on cabbage plants in the laboratory (*ca*. 25°C, 70% relative humidity, under a 16 hr light: 8 hr dark cycle, unless otherwise indicated) until pupation. Pupae were kept in a cage for eclosion. Adult butterflies were fed with 10% honey water. The colony was replenished annually with field-collected butterflies every two months.

#### Clawed frog rearing

The female African clawed frog *Xenopus laevis* were purchased from Haiwei Panshi Biomedical Technology Co., Ltd, Qingdao, China, and reared on pork liver at *ca*. 18°C in the Laboratory Animal Center, Institute of Genetics and Developmental Biology, Chinese Academy of Sciences.

#### Fly husbandry

The fruit fly *Drosophila melanogaster* was reared on standard cornmeal-yeast-agar medium and kept under standard conditions (*ca*. 25°C, 12 hr light: 12 hr dark cycle). *Gr5a-GAL4*;*Dr/*sb fly was obtained from the Bloomington Drosophila Stock Center. An isogenized strain of w^1118^ was used as a wild-type control.

### Chemical sources

Sinigrin hydrate, vanillic acid, amygdalin, L-canavanine, phloridzin dihydrate, umbelliferone, rutin hydrate, salicin, gallic acid, naringin, sinapic acid, gramine, caffeine, sucrose, allyl isothiocyanate, coumarin, methyl jasmonate, brilliant blue FCF and sulforhodamine B were purchased from Sigma-Aldrich (St. Louis, MO, USA). Glucoiberin potassium salt and glucobrassicin potassium salt were purchased from Extrasynthese (Lyon, France). Gluconapin potassium salt and gluconasturtiin potassium salt were purchased from ChromaDex (Irvine, CA, USA). Quinine, chloroquine, denatonium and indole were purchased from Aladdin (Shanghai, China). Salicylic acid and jasmonic acid were purchased from TCI (TCI Shanghai, China). Berberine was purchased from Macklin (Shanghai, China).

### Feeding choice test

Fresh cowpea foliage was used as a substrate for testing the effects of glucosinolates on feeding of *P*. *rapae* larvae [16]. Two choice behavior assays were performed as previously described with some modifications [17]. Briefly, four discs 1.5 cm in diameter from the same leaf were placed in one Petri dish of 9.0 cm diameter. The upper surface of each disc was supplied with 20 μL of glucosinolates dissolved in water (treated disc) or the same volume of water (control disc). The concentration gradients of each glucosinolate ranged from 10^−6^ to 10^−2^ M in bioassays. Treated and control discs were alternately placed. The fifth instar larvae were placed in the center of the Petri dish. The inside of Petri dish lid was covered with wet filter paper to keep humidity. When the total feeding area was larger than 25% or after 24 h feeding, the area of each disc consumed by larvae was calculated, and feeding preference was estimated as the preference index (PI): PI = (consumed area of the treated disc) / total consumed area (consumed area of the treated disc + consumed area of the control disc).

### Tip recording

The tip recording technique was used to record the electrophysiological responses of the larval sensilla styloconica to glucosinolates following a previously described method [21,72]. To avoid possible adaptation and reduced sensitivity of tested sensilla, the interval between two stimulations was at least three min. The spikes were classified and counted from the first 1000 ms after stimulation using Autospike v.3.7 software (Syntech, Hilversum, the Netherlands). All tested glucosinolates were dissolved in 2 mM KCl solution and the 2 mM KCl solution was tested as the control. The sensilla styloconica on larval maxilla were first randomly stimulated by sinigrin, gluconapin, glucoiberin, glucobrassicin and gluconasturtiin at 10 mM. Concentrations of 0.01 mM, 0.1 mM, 1.0 mM and 10 mM for each compound were used in dose-response experiments.

A modified tip recording protocol was used to record the response from tarsal taste sensilla in adults [19]. Briefly, two to three days old virgin adult butterflies were decapitated and wings, abdomen and meso- and meta-thoracic legs were removed. The reference electrode was inserted into the thorax and connected to the input of a pre-amplifier. The distal part of foreleg was fixed on a small platform with double sided adhesive tape, and the ventral side of the tarsi was exposed. The sensillum recorded from was chosen randomly among lateral and medial tarsal sensilla. The tested glucosinolates and their concentrations were the same as for larval recording described above.

### Transcriptome sequencing

Foreleg tarsi of male and female *P*. *rapae* adults and larval mouthparts were collected and quickly frozen in liquid nitrogen, and then stored at -80 ^o^C for transcriptome sequencing. Every tissue was prepared for three biological replicates. Total RNA was isolated using the RNeasy Plus Universal Mini Kit (QIAGEN, Hilden, Germany). The cDNA library construction and Illumina sequencing were performed by Illumina HiSeq4000 platform sequencing at Novogene Co., Ltd., Beijing, China. Paired-end reads were generated using a PE150 strategy. High quality clean data (clean reads) were obtained by removing reads containing adapter, poly-N (empty reads) and low quality reads (N > 10% sequences) from raw data. Transcriptome assembly was accomplished using Trinity v2.4.0 with min_kmer_cov set to 2 by default and all other parameters set default [73]. The annotation of GRs was accomplished by BLASTx searching against Nr database (NCBI non-redundant protein sequences) and Swiss-Prot database (a manually annotated and reviewed protein sequence database) with e values < 1e-5. The TPM values of candidate *GR* genes were estimated to indicate the tissue abundance distribution of *GR* genes by RSEM v1.2.15 software [74]. The open reading frames (ORFs) were predicted by ORF finder (https://www.ncbi.nlm.nih.gov/orffinder/).

### Phylogenetic analysis

To compare the evolutionary relationship of GRs, a phylogenetic tree was constructed with the GR sequences from *P*. *rapae* and other Lepidoptera species, including *Bombyx mori*, *Heliconius melpomene* [43,44]. Amino acid sequences were aligned with MAFFT v7.455 [75], gap sites were removed with trimAl v1.4 [76] and Maximum likelihood phylogenies were inferred using IQ-TREE v1.6.8 [77] under the Jones-Taylor-Thornton (JTT) + F + G4 model for 5000 ultrafast bootstraps. Phylogenetic tree was visualized and graphically edited in FigTree v1.4.4 (http://tree.bio.ed.ac.uk/software/figtree/).

### Quantitative real-time PCR (qRT-PCR)

Total RNA was obtained by the RNeasy Plus Universal Mini Kit (QIAGEN, Hilden, Germany) and cDNA was also prepared using M-MLV Reverse Transcriptase (Promega, Wisconsin, WI, USA) following the manufacturer’s protocols. qRT-PCR was performed on a QuantStudio 3 Real-Time PCR System (Thermo Fisher Scientific, Waltham, MA, USA) using SYBR *Premix Ex Taq* II (Tli RNaseH Plus; TaKaRa, Shiga, Japan). The specific primer sequences were listed in S2 Table. The relative expression levels of target genes were calculated according to the 2^–ΔΔCt^ method [78]. *Elongation factor 1* (*EF1*, GenBank No. XM_022262780.1) was used as reference gene.

### Functional analysis of PrapGrs

The full-length coding sequences of *PrapGr28* and *PrapGr15* were cloned into pGEM-T easy vector (Promega, Madison, WI, USA), and then subcloned into pCS2+ vector. The primer sequences were listed in S2 Table. The recombinant pCS2+ vectors were linearized by restriction enzyme *Not* I (TaKaRa, Shiga, Japan), and cRNAs were synthesized from the linearized recombinant pCS2+ vectors with mMESSAGE mMACHINE SP6 Transcription Kit (Ambion, Austin, TX, USA). Purified cRNAs were re-suspended in RNase-free water at a concentration of 2 mg/mL and stored at -80°C.

The acquisition of *X*. *laevis* oocytes was performed following a previously described protocol [79]. *X*. *laevis* was anesthetized by bathing in ice water for 30 min. Oocytes were surgically collected and treated with 2 mg/mL of collagenase type I in washing buffer for *ca*. 1–2 h at room temperature. Mature healthy oocytes were microinjected with 27.6 nL of *PrapGr28* cRNA, *PrapGr15* cRNA and *PrapGr28*/*PrapGr15* (mixtures with the ratio of 1:1) cRNA, respectively. The oocytes were parallelly injected with water as control. Injected oocytes were incubated for 4–6 days at 16°C in Barth’s solution supplemented with 5% dialyzed horse serum, 50 mg/mL tetracycline, 100 mg/mL streptomycin and 550 mg/mL sodium pyruvate.

Two-electrode voltage clamp technique was employed to record whole-cell currents of the oocytes responding to the chemicals [62]. The concentration of 1.0 mM for each chemical was randomly used at first, and then concentration gradients (ranging from 10^−4^ M, 5×10^−4^ M, 10^−3^ M, 5×10^−3^ M, 10^−2^ M, 5×10^−2^ M) were recorded later when a clear current response was detected. Intracellular glass electrodes were filled with 3 M KCl and presented resistances of 0.2–2.0 MΩ. Signals were amplified with an OC-725C amplifier (Warner Instruments, Hamden, CT, USA) at a holding potential of -80 mV, low-pass filtered at 50 Hz and digitized at 1 kHz. Data acquisition and analysis were carried out with Digidata 1322A and pCLAMP software (Axon Instruments Inc., Foster City, CA, USA).

### *In situ* hybridization

Two-color *in situ* hybridization was performed using a previously described method [62]. The gene-specific probe sequences of *PrapGr28* and *PrapGr15* were amplified with specific primers and labeled using Biotin (Bio) RNA Labeling Mix (Roche, Mannheim, Germany) and Digoxigenin (Dig) RNA Labeling Kit (SP6/T7) (Roche, Mannheim, Germany), respectively. The primer sequences were listed in S2 Table. The RNA probes were subsequently fragmented to a length of about 300 bp by incubating in carbonate buffer.

The tarsi were dissected from two to three day-old adult butterflies, and then embedded in JUNG tissue freezing medium (Leica, Nussloch, Germany). After that, the samples were cut into 12 μm slices at -22°C by using a freezing microtome (Leica M1950, Germany). The procedures of hybridization were conducted according to a previously described method [62]. Briefly, after fixing and washing steps, 100 mL hybridization solution (Boster, Wuhan, China) containing both Dig and Bio probes was added to the tissue sections. After adding a coverslip, slides were incubated in a humid box at 55°C overnight. After hybridization, slides were washed twice for 30 min in 0.1 × saline sodium citrate (SSC) at 60°C, treated with 1% blocking reagent (Roche, Mannheim, Germany) in Tris-buffered saline (TBS) with 0.05% Tween-20 (Tianma, Beijing, China) (TBST) for 30 min at room temperature, and then incubated for 60 min with anti-digoxigen (Roche, Mannheim, Germany) and Strepavidin-HRP (PerkinElmer, Boston, USA). Visualization of hybridization signals was performed by incubating the sections first for 30 min with HNPP/Fast Red (Roche, Mannheim, Germany), followed by three 5 min washes in TBST at room temperature with shaking. The sections were incubated with Biotinyl Tyramide Working Solution for 8 min at room temperature followed by the TSA Fluorescein System protocols (PerkinElmer, Boston, USA). Sections were then washed three times for 5 min each in TBST at room temperature with shaking. Finally, sections were mounted in Antifade Mounting Medium (Beyotime, Beijing, China). Pictures were taken with a confocal microscope (Zeiss LSM710, Oberkochen, Germany).

### Transgenic *Drosophila* construction and test

The *PrapGr28* full-length coding DNA sequence (CDS) was constructed into the *p10* plasmid (*pJFRC-28-10-10×UAS-IVS-GFP-P10*, add gene plasmid # 36431). For phiC31 integrase-mediated transformation on chromosome 3, *p10-PrapGr28* plasmids were injected into attp2 fly embryos (*P{y[+t7*.*7] = nos-phiC31\int*.*NLS}X*, *y[1] sc[1] v[1] sev[21]*; *P{y[+t7*.*7] = CaryP}attP2*, BDSC # 25710) by custom injection service provided by Qidong Fungene Biotechnology (Jiangsu Province, China) to generate transformant *UAS*-*PrapGr28* fly line for further crossings. *UAS*-*PrapGr28* flies were crossed with *Gr5a-GAL4* lines (genotype: *w*; *Gr5a-GAL4*;*Dr/*sb, BDSC # 57591) to generate *Gr5a*-*GAL4*/Cyo;*UAS*-*PrapGr28*/*Dr* flies. The *Gr5a*-*GAL4*;*UAS*-*PrapGr28* homozygotes were obtained by sibling crosses. The labella of flies were collected for the detection of *PrapGr28* expression by RT-PCR. *Tubulin* (GenBank No. NM_057424.4) was used as reference gene. The primer sequences were listed in S2 Table. Finally, all the flies maintained for tip recordings and behavioral experiments as follows.

The tip recording of L-type sensilla on the labial palp was performed as previously described with some modifications [34,80]. Briefly, a glass capillary filled with Ringer’s solution was inserted into the fly abdomen all the way through to the head as the reference electrode. A glass capillary of 10–15 μm tip diameter were filled with stimulus solutions as the recording electrode. All recording procedures in this experiment are the same as described for larval recording in *P*. *rapae*. Glucosinolates were dissolved in 1 mM KCl solution and the 1 mM KCl solution was set as a control. The sensilla were first randomly stimulated by 10 mM of sinigrin, gluconapin, glucoiberin, glucobrassicin and gluconasturtiin. The concentrations of 0.1 mM, 1.0 mM and 10 mM were then used for each compound in dose-response experiments. 10 mM sucrose was used as the positive control to determine the correctness of the tested sensilla.

In the flies, the binary food-choice assays were measured following the protocol reported previously [34,46,80]. Briefly, 40–50 flies (3–7 days old) were collected under CO_2_ anesthesia and starved for 6–9 hr in vials at room temperature, and then the flies were introduced into a box containing 8-strip tube caps filled with control and compounds in alternate wells. For the control, the wells only contained 1% agarose were mixed with a blue dye (brilliant blue FCF, 0.125 mg/mL). For the treatment, the wells containing 1% agarose plus 10 mM sinigrin were mixed with a red dye (0.2 mg/mL sulforhodamine B). The dyes were used to monitor the food intake for the flies. Flies were allowed to freely feed overnight in a dark room at room temperature. Subsequently, all flies were anesthetized at -20°C for scoring the flies to calculate preference index (PI) by using the following equation: PI = (number of red abdomens + ½ the number of purple abdomens)/ total number of fed flies.

### dsRNA synthesis and injection

Total RNA and cDNA of female tarsi were obtained as described above. To synthesize the *PrapGr28* dsRNA, region a and b (Fig 8A) were first amplified by specific primers, and then cloned into *pEASY*-T1vector (TransGen Biotech, Beijing, China). After sequencing, positive clone plasmids were used as PCR templates to acquire novel PCR products using the primers containing T7 promoter. These acquired PCR products were used as the template for dsRNA synthesis. The primer sequences were listed in S2 Table. dsRNAs were prepared by T7 RiboMAX Express RNAi System (Promega, Madison, WI, USA) following the manufacturer’s protocol. *GFP* (green fluorescent protein, GenBank No. AAX31732.1) dsRNA was parallelly synthesized as control. The dsRNA was diluted to 2000 ng/μL and stored at -80°C until used.

Other than *PrapGr28* a dsRNA and *PrapGr28* b dsRNA, a mixture of *PrapGr28* a+b dsRNA was also injected to increase the RNAi effectiveness. In the groups of *GFP* dsRNA, *PrapGr28* a dsRNA, and *PrapGr28* b dsRNA, each pupa was injected with 2.5 μL dsRNA; in the *PrapGr28* a+b dsRNA group, each pupa was injected with 5 μL dsRNA, with 2.5 μL *PrapGr28* a and 2.5 μL *PrapGr28* b dsRNA. All dsRNAs were injected into female pupa 3 days before eclosion, using a microliter syringe (Hamilton, Bonaduz, Switzerland) [39]. After injection, pupae were placed at 28°C until eclosion. The expression level of *PrapGr28* was verified by qRT-PCR, for which wild type and *GFP* dsRNA group were used as control. The primer sequences were listed in S2 Table. The sensilla on the fifth foreleg tarsi in female butterflies injected with dsRNA were first stimulated by 10 mM of sinigrin, gluconapin, glucoiberin, glucobrassicin and gluconasturtiin. The concentrations of 0.1 mM, 1.0 mM and 10 mM were then used for each compound in dose-response experiments.

### Statistical analysis

Statistical analyses were performed using SPSS 20.0 (IBM Inc., Chicago, IL, USA) and GraphPad Prism 5 (GraphPad Software, Inc., La Jolla, CA, USA). *n* represents the replicate number. The paired Student’s *t*-test was used to evaluate the feeding preference to glucosinolates. The data of electrophysiological responses, expression level of *GRs*, and two-electrode voltage-clamp recording were analyzed by one-way ANOVA for analysis of variance and compared with Tukey HSD test. Data are presented as mean ± SEM. Different letters indicate significant differences. Asterisks indicate statistical significance (*P < 0.05, ** *P* < 0.01, *** *P* < 0.001). The raw data of the figures and statistical analyses in this study are provided in S3 Table.

## Supporting information

S1 Fig Representative images showing the feeding preference of *P. rapae* larvae to glucosinolates. (**A**) sinigrin, (**B**) gluconapin, (**C**) glucoiberin, (**D**) glucobrassicin, and (**E**) gluconasturtiin treated leaf discs with a series of concentrations. The single larva was removed from the Petri dish when 25% of the total leaf disk area was consumed, or larva was fed for 24 h.
(TIF)

S2 Fig Electrophysiological activity recorded from sensilla styloconica on larval maxilla of *P. rapae* to glucosinolates. Example of response from lateral sensilla styloconica (**A**-**E**) and medial sensilla styloconica (**F**). Two millimolar KCl was used as control.
(TIF)

S3 Fig Electrophysiological activity recorded from taste sensilla on the fifth foreleg-tarsi of female *P. rapae* to glucosinolates. Example of response of lateral tarsal sensilla (**A**, **B**) and medial tarsal sensilla (**C**-**G**). Two millimolar KCl was used as control.
(TIF)

S4 Fig Spike sorting in the tarsal medial sensilla of *P. rapae* adults to glucosinolates. The spikes of sample recordings from the tarsal medial sensilla of female (**A**) and male (**B**) adults stimulated by sinigrin, gluconapin, glucoiberin, glucobrassicin, and gluconasturtiin at 10 mM were sorted based on the amplitude. Asterisk and triangle represent the smaller and larger amplitude spikes, respectively.
(TIF)

S5 Fig Electrophysiological activity recorded from taste sensilla on the fifth foreleg-tarsi of male *P. rapae* to glucosinolates. Example of response from lateral tarsal sensilla (**A**, **B**) and medial tarsal sensilla (**C**-**G**). Two millimolar KCl was used as control.
(TIF)

S6 Fig TPM (transcripts per kilobase of exon model per million mapped reads) values of candidate *GR* genes. *n* = 3. Data are presented as mean ± SEM. One-way ANOVA with Tukey HSD test was used. * *P* < 0.05, ** *P* < 0.01, *** *P* < 0.001, compared with female tarsi.
(TIF)

S7 Fig Secondary structure prediction and sequence alignment of bitter receptors. (**A**, **B**) The predicted secondary structure of (**A**) PrapGr28 and (**B**) PrapGr15. The image was constructed by TOPO2 software (http://www.sacs.ucsf.edu/TOPO2/) based on the secondary structure predicted by TOPCONS (topcons.net) models. The model with a reliable seven-transmembrane structure was adopted. (**C**) Similarity analysis of PrapGr28, PrapGr15 and the other putative bitter receptors. The homology analysis of putative bitter receptors in *P*. *rapae* were performed by multiple sequence alignment using the DNAMAN software.
(TIF)

S8 Fig Inward current responses of *Xenopus* oocytes expressing taste receptors to compounds. Representative image of inward current responses of *Xenopus* oocytes expressing *PrapGr28* (**A**) and *PrapGr15* (**B**) in response to compounds at 1 mM.
(TIF)

S9 Fig Functional analysis of *Xenopus* oocytes expressing *PrapGr28/PrapGr15* to compounds. (**A**) Inward current response and (**B**) response profiles of *Xenopus* oocytes expressing *PrapGr28*/*PrapGr15* in response to compounds at 1 mM. *n* represents the number of oocytes and are labeled in the figures. (**C**) Inward current responses and (**D**) dose-response curve of *Xenopus* oocytes expressing *PrapGr28*/*PrapGr15* (*n* = 5) stimulated with a range of sinigrin concentrations. Data are presented as mean ± SEM. Different letters labeled indicate significant differences. One-way ANOVA with Tukey HSD test was used.
(TIF)

S10 Fig Two-electrode voltage-clamp recordings of *Xenopus* oocytes injected with water. (**A**) Inward current responses and (**B**) response profiles of *Xenopus* oocytes injected with water in response to compounds at 1 mM. *n* = 4–9. Data are presented as mean ± SEM. One-way ANOVA with Tukey HSD test was used.
(TIF)

S11 Fig Response properties of taste sensilla on the labellum of *D. melanogaster* to sucrose. (**A**) Representative traces and (**B**) spike frequencies of L-type sensillum on the fly labellum in response to 10 mM sucrose. *n* = 3–4. Data are presented as mean ± SEM. One-way ANOVA with Tukey HSD test was used for comparison with control flies.
(TIF)

S12 Fig Examples of responses of L-type sensilla in the w^1118^, *Gr5a-GAL4;Dr/sb, Cyo/sp;UAS-PrapGr28*, and *Gr5a-GAL4;UAS-PrapGr28* fly lines to gluconapin, glucoiberin, glucobrassicin, and gluconasturtiin at 10 mM. (TIF)

S13 Fig Firing patterns of taste sensilla on the *D. melanogaster* labellum to sinigrin. Example of response of L-type sensilla in the w^1118^, *Gr5a-GAL4*;*Dr*/sb, *Cyo*/sp;*UAS*-*PrapGr28*, and *Gr5a*-*GAL4*;*UAS*-*PrapGr28* fly lines to different concentrations of sinigrin.
(TIF)

S14 Fig Feeding preference of *D. melanogaster* expressing *PrapGr28* to sinigrin. The presence of PrapGr28 reduced the aversive behavior to 10 mM sinigrin in the *Gr5a*-*GAL4*;*UAS*-*PrapGr28* line. The w^1118^, *Gr5a-GAL4*;*Dr*/sb, and *Cyo*/sp;*UAS*-*PrapGr28* fly lines were used as control lines. *n* = 10–11. Forty to fifty flies were used for each replicate. Data are presented as mean ± SEM. One-way ANOVA with Tukey HSD test was used. ** *P* < 0.01, compared with control flies.
(TIF)

S15 Fig Location of taste receptors in the foreleg tarsi of *P. rapae* adults. Co-expression patterns of *PrapGr28* and *PrapGr15* in female (**A**) and male (**B**) *P*. *rapae* adult foreleg tarsi. *PrapGr28* antisense RNA probe was biotin-labeled and visualized by green fluorescence. *PrapGr15* antisense RNA probe was digoxigenin-labeled and visualized by red fluorescence. The dashed frame areas are enlarged and shown on the right. Arrows show labelled somata with probes synthesized from targeted genes. Bright-field images are presented as references.
(TIF)

S16 Fig Firing patterns of taste sensilla on the foreleg-tarsi of *PrapGr28* knockdown butterflies to glucoiberin, glucobrassicin, and gluconasturtiin at 10 mM. (TIF)

S17 Fig Examples of responses from medial tarsal sensilla stimulated with different concentrations of sinigrin (A) and gluconapin (B). (TIF)

S18 Fig Firing patterns of lateral sensilla on the foreleg-tarsi of *PrapGr28* knockdown butterflies to glucosinolates. (**A**) Typical electrophysiological responses and (**B**) spike frequencies of lateral tarsal sensilla in response to 10 mM glucobrassicin (*n* = 4–8) and gluconasturtiin (*n* = 6–10). Data are presented as mean ± SEM. One-way ANOVA with Tukey HSD test was used for comparison with the control of *GFP* dsRNA.
(TIF)

S1 Table Candidate GRs in *P. rapae*. (DOCX)

S2 Table Primers used in this study. (DOCX)

S3 Table Raw data used in the figures and statistical analyses. (XLSX)
