# Supplementary figures and images for: Identification of a gustatory receptor tuned to sinigrin in the cabbage butterfly Pieris rapae
Source: PLoS Genet. 2021 Jul 15;17(7):e1009527. doi: 10.1371/journal.pgen.1009527 (PMC8282186; doi:10.1371/journal.pgen.1009527)

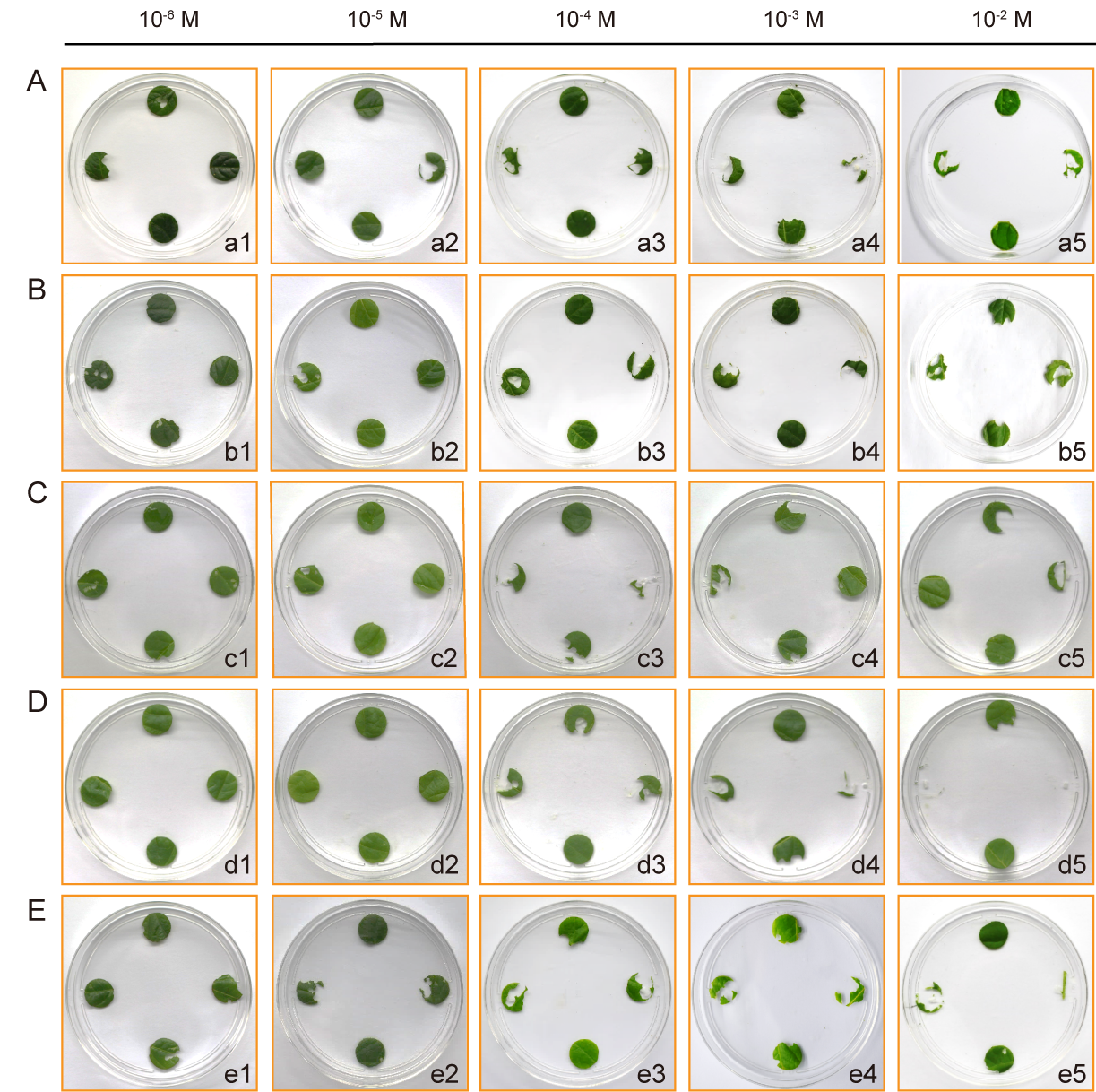

Supplement: S1 Fig — (A) sinigrin, (B) gluconapin, (C) glucoiberin, (D) glucobrassicin, and (E) gluconasturtiin treated leaf discs with a series of concentrations. The single larva was removed from the Petri dish when 25% of the total leaf disk area was consumed, or larva was fed for 24 h. (TIF) [file pgen.1009527.s001.tif]

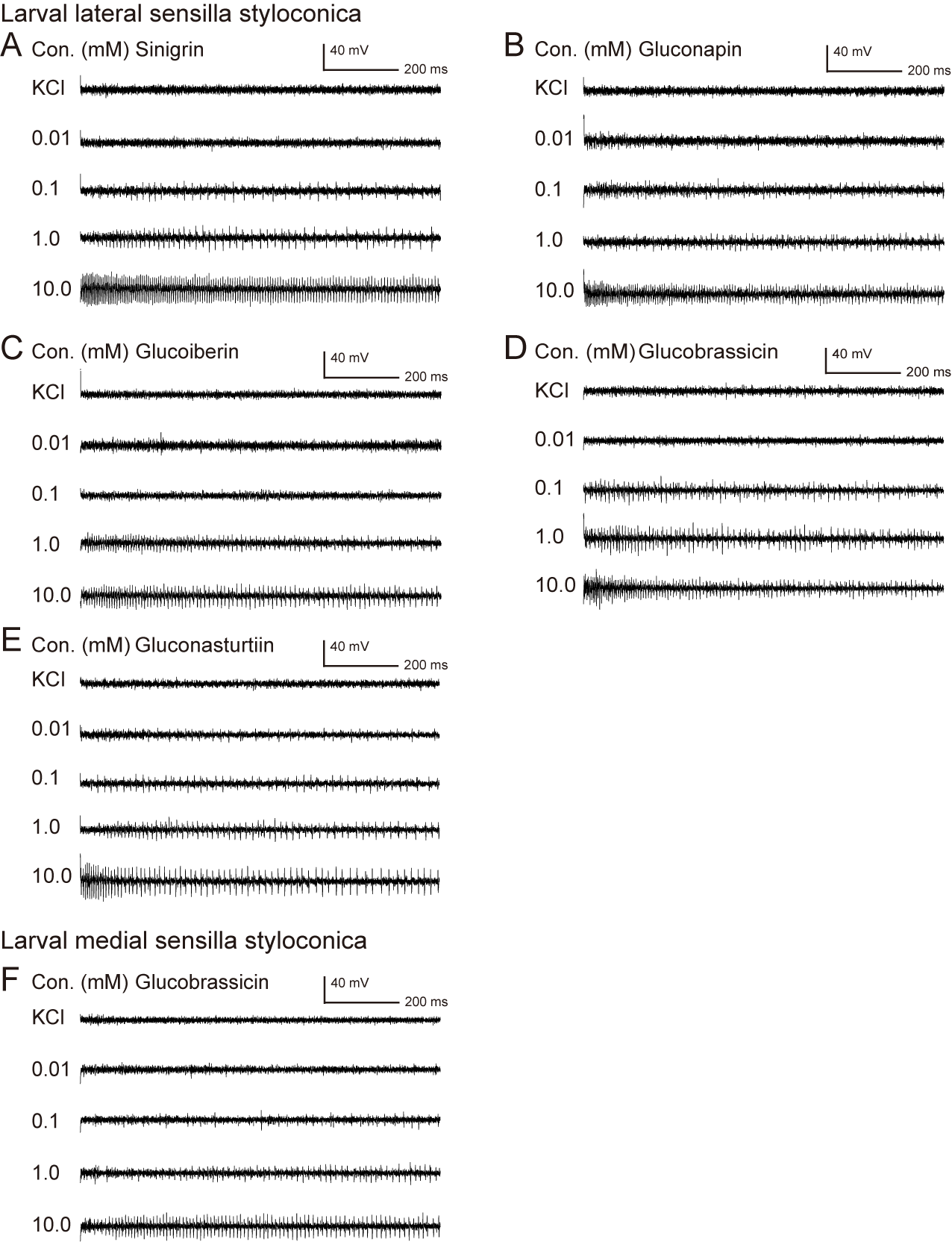

Supplement: S2 Fig — Example of response from lateral sensilla styloconica (A-E) and medial sensilla styloconica (F). Two millimolar KCl was used as control. (TIF) [file pgen.1009527.s002.tif]

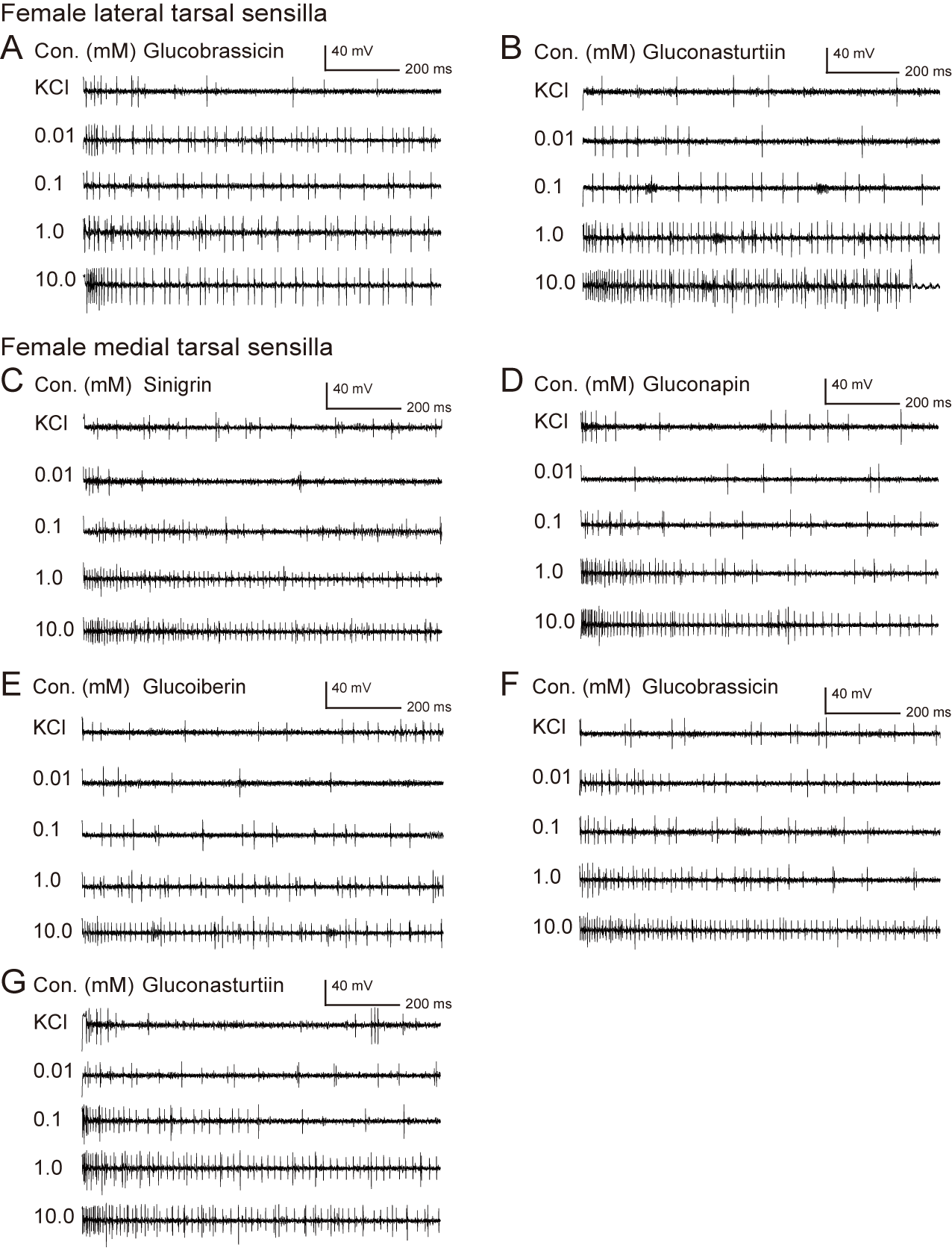

Supplement: S3 Fig — Example of response of lateral tarsal sensilla (A, B) and medial tarsal sensilla (C-G). Two millimolar KCl was used as control. (TIF) [file pgen.1009527.s003.tif]

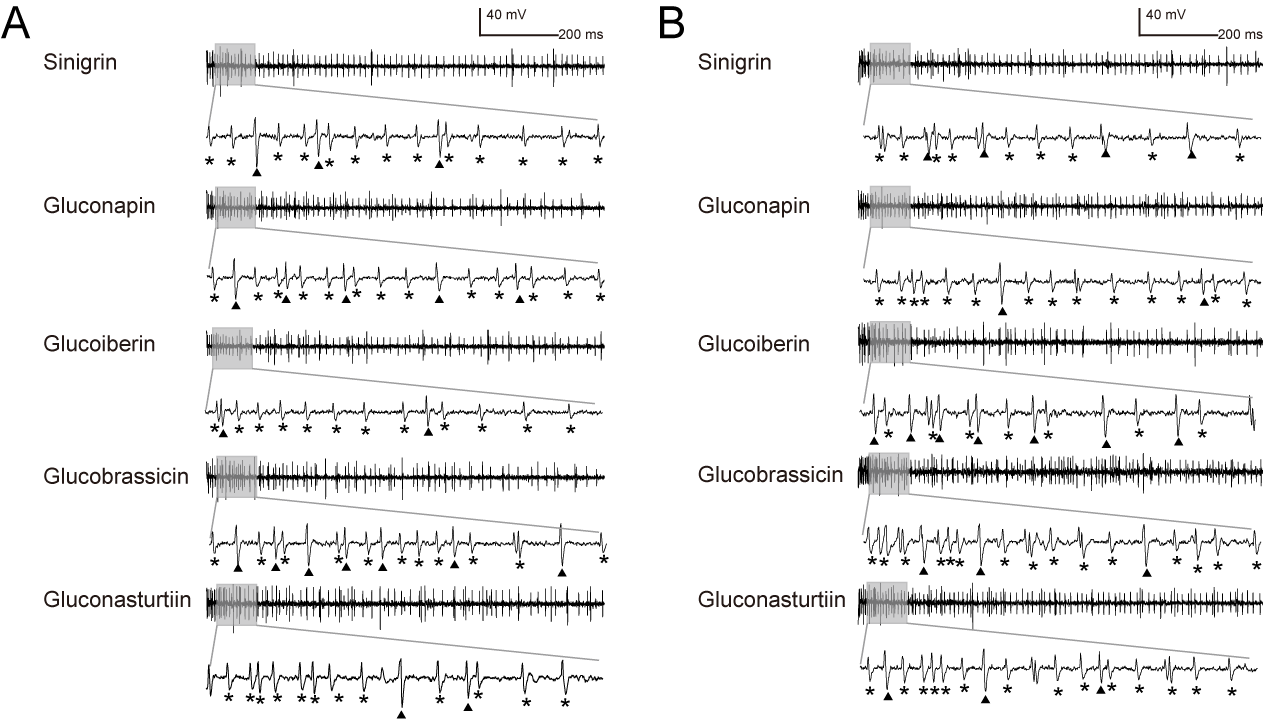

Supplement: S4 Fig — The spikes of sample recordings from the tarsal medial sensilla of female (A) and male (B) adults stimulated by sinigrin, gluconapin, glucoiberin, glucobrassicin, and gluconasturtiin at 10 mM were sorted based on the amplitude. Asterisk and triangle represent the smaller and larger amplitude spikes, respectively. (TIF) [file pgen.1009527.s004.tif]

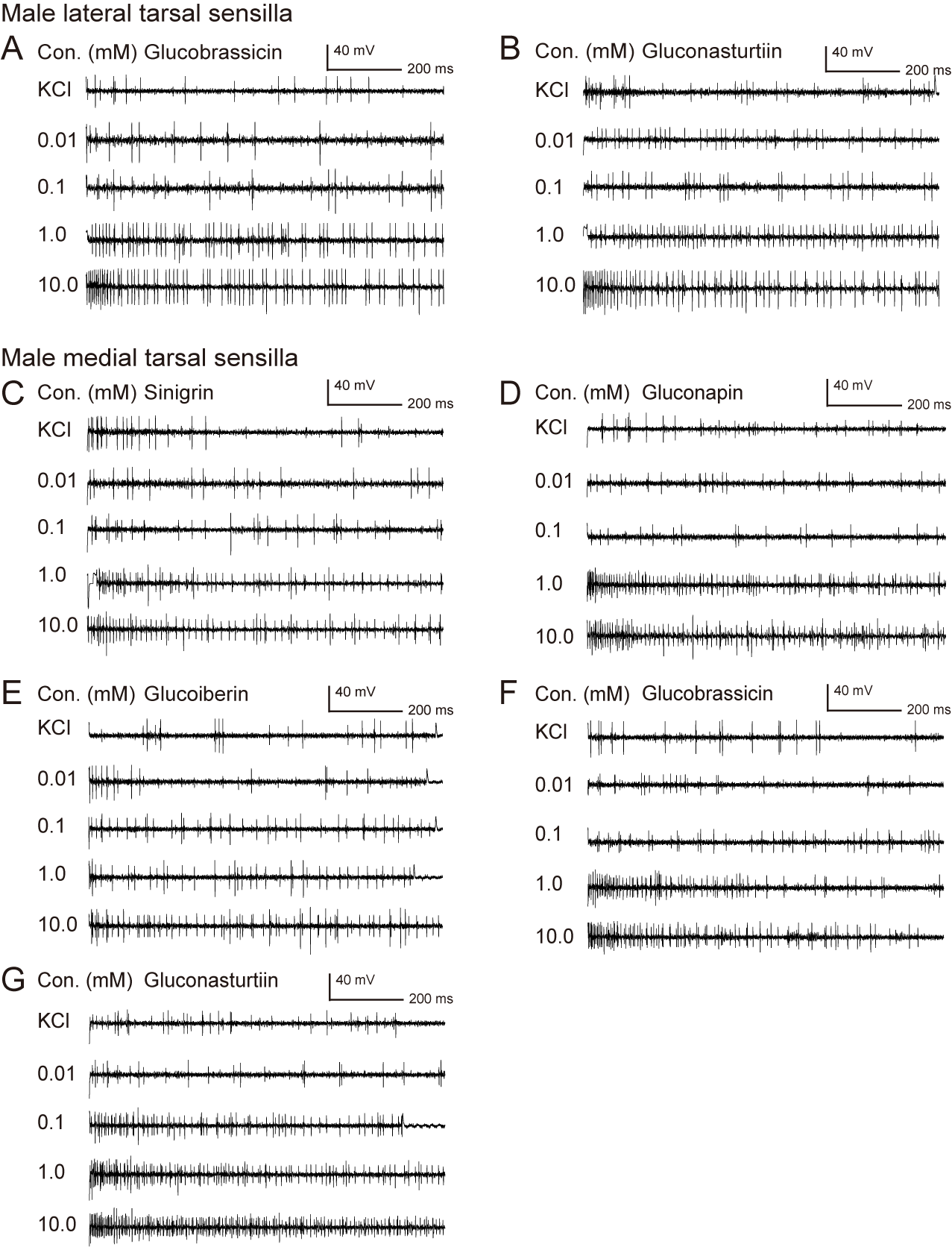

Supplement: S5 Fig — Example of response from lateral tarsal sensilla (A, B) and medial tarsal sensilla (C-G). Two millimolar KCl was used as control. (TIF) [file pgen.1009527.s005.tif]

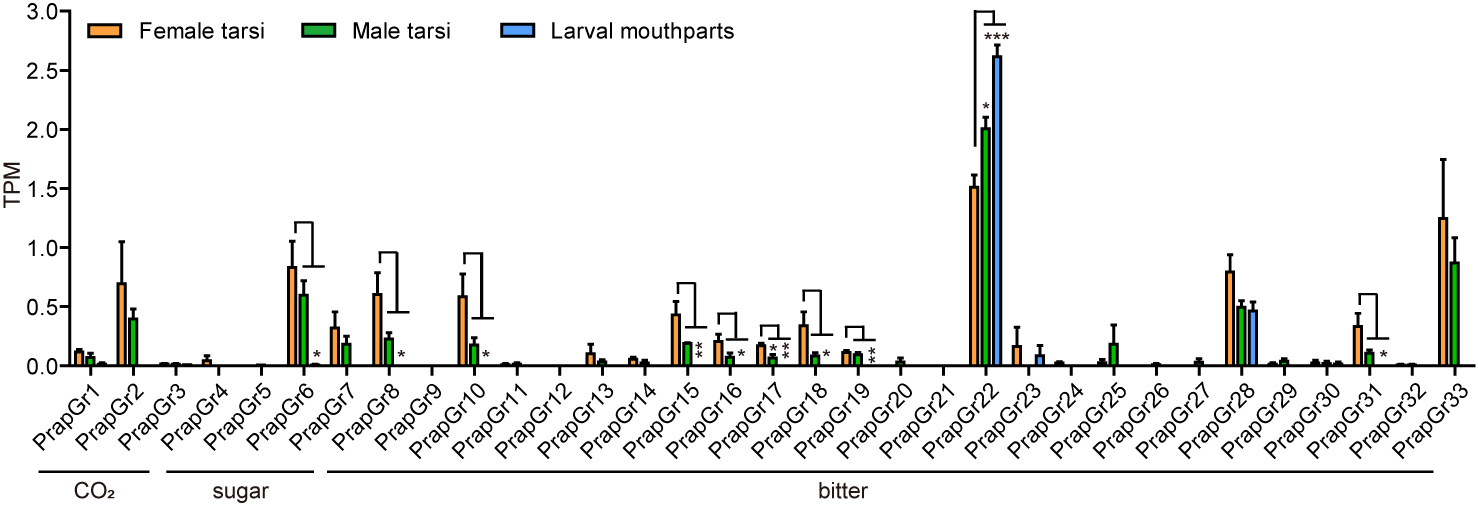

Supplement: S6 Fig — n = 3. Data are presented as mean ± SEM. One-way ANOVA with Tukey HSD test was used. * P < 0.05, ** P < 0.01, *** P < 0.001, compared with female tarsi. (TIF) [file pgen.1009527.s006.tif]

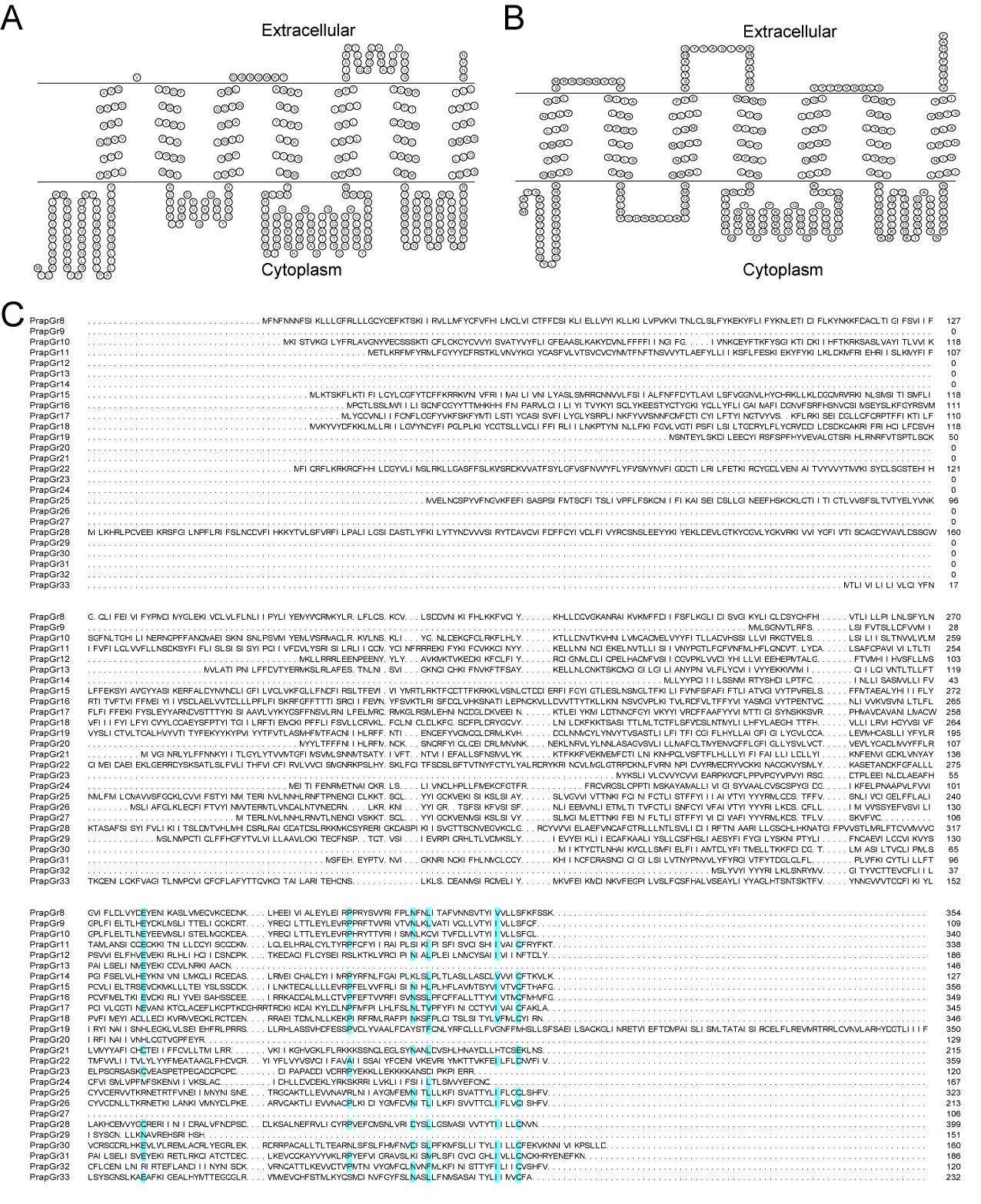

Supplement: S7 Fig — (A, B) The predicted secondary structure of (A) PrapGr28 and (B) PrapGr15. The image was constructed by TOPO2 software (http://www.sacs.ucsf.edu/TOPO2/) based on the secondary structure predicted by TOPCONS (topcons.net) models. The model with a reliable seven-transmembrane structure was adopted. (C) Similarity analysis of PrapGr28, PrapGr15 and the other putative bitter receptors. The homology analysis of putative bitter receptors in P. rapae were performed by multiple sequence alignment using the DNAMAN software. (TIF) [file pgen.1009527.s007.tif]

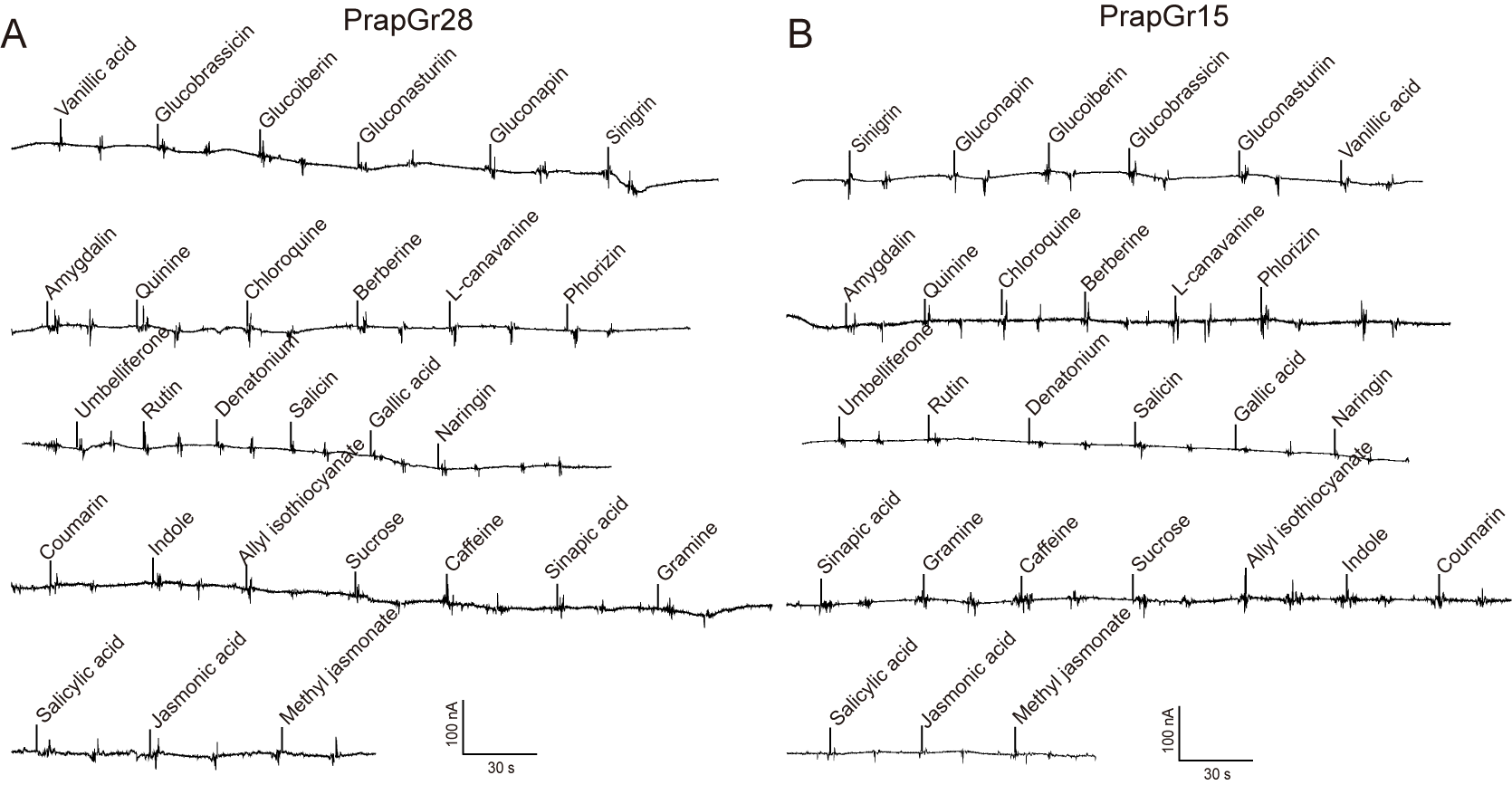

Supplement: S8 Fig — Representative image of inward current responses of Xenopus oocytes expressing PrapGr28 (A) and PrapGr15 (B) in response to compounds at 1 mM. (TIF) [file pgen.1009527.s008.tif]

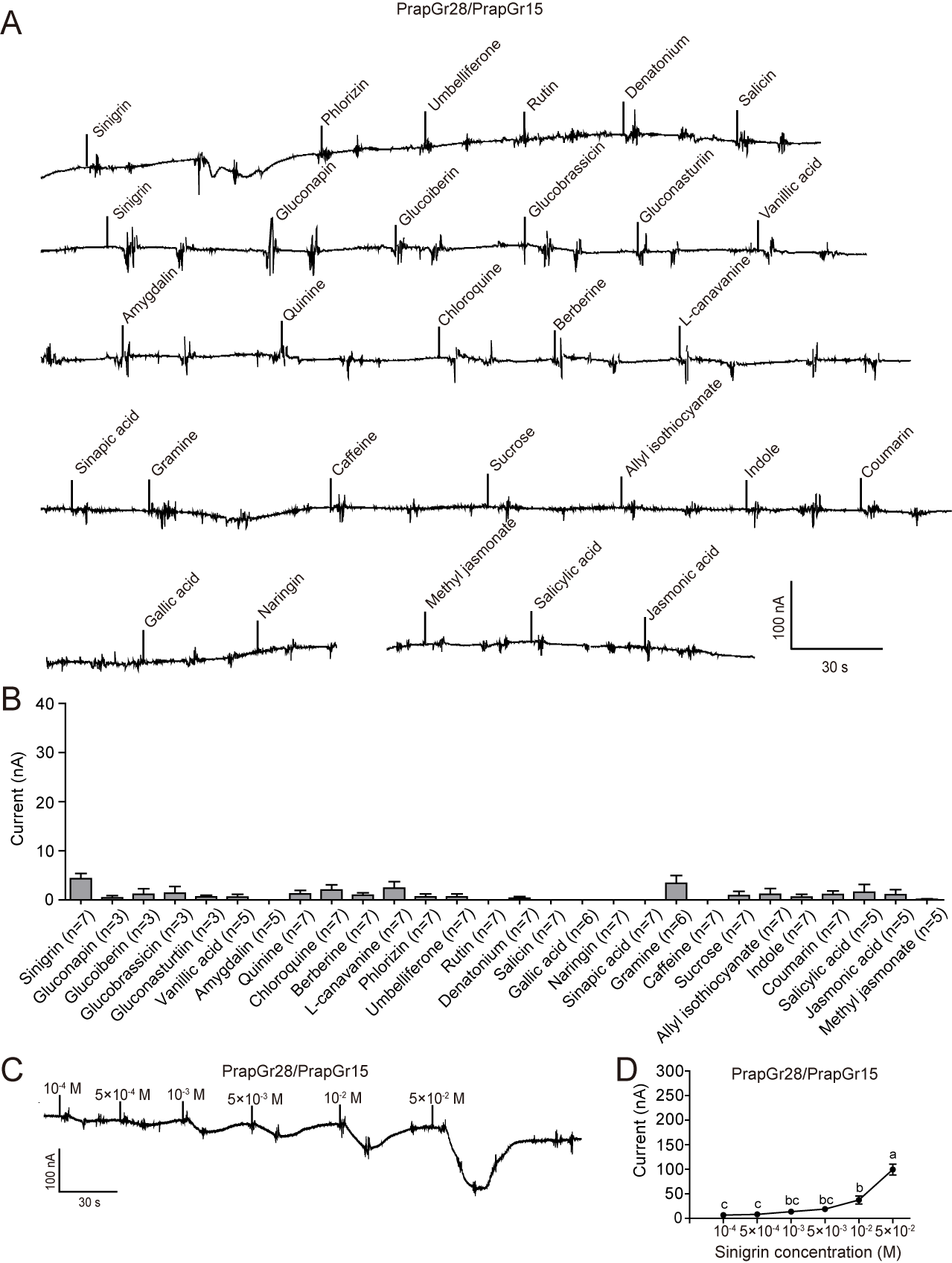

Supplement: S9 Fig — (A) Inward current response and (B) response profiles of Xenopus oocytes expressing PrapGr28/PrapGr15 in response to compounds at 1 mM. n represents the number of oocytes and are labeled in the figures. (C) Inward current responses and (D) dose-response curve of Xenopus oocytes expressing PrapGr28/PrapGr15 (n = 5) stimulated with a range of sinigrin concentrations. Data are presented as mean ± SEM. Different letters labeled indicate significant differences. One-way ANOVA with Tukey HSD test was used. (TIF) [file pgen.1009527.s009.tif]

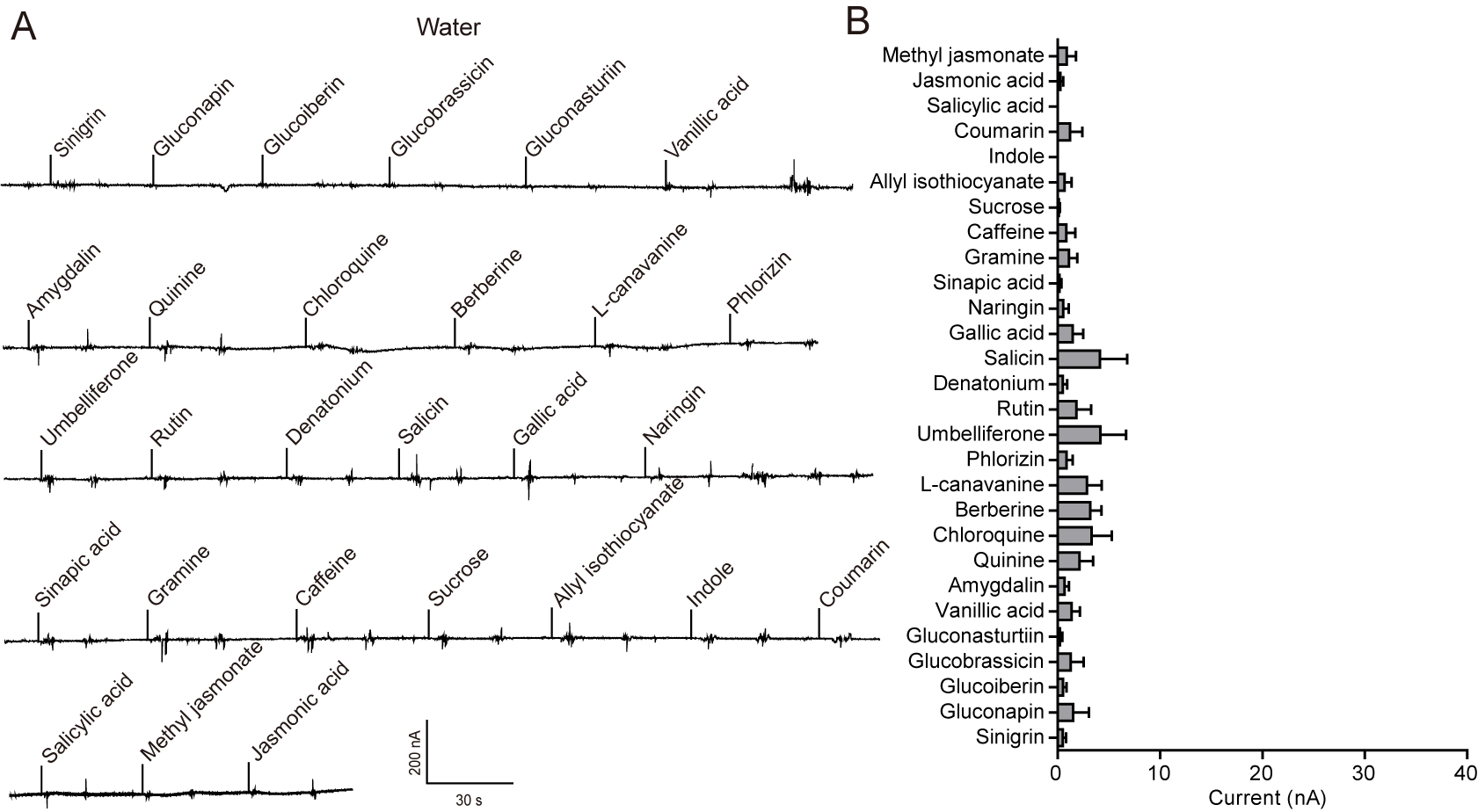

Supplement: S10 Fig — (A) Inward current responses and (B) response profiles of Xenopus oocytes injected with water in response to compounds at 1 mM. n = 4–9. Data are presented as mean ± SEM. One-way ANOVA with Tukey HSD test was used. (TIF) [file pgen.1009527.s010.tif]

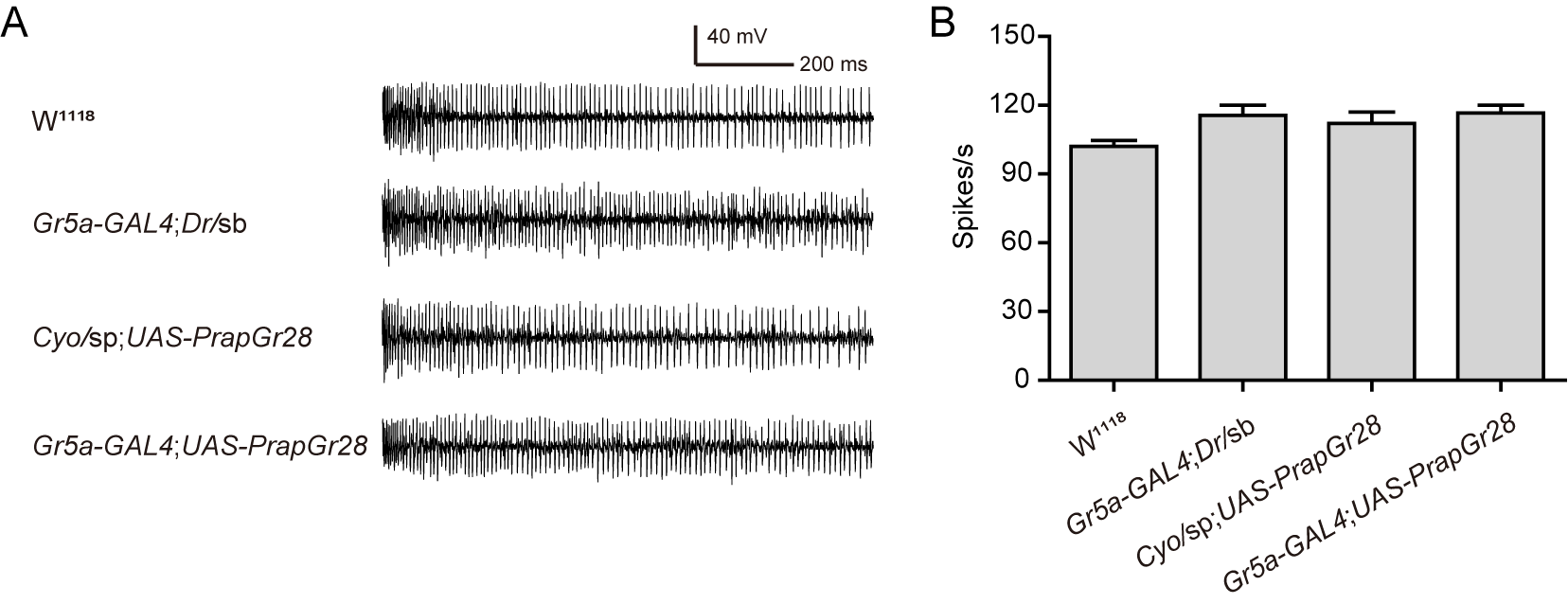

Supplement: S11 Fig — (A) Representative traces and (B) spike frequencies of L-type sensillum on the fly labellum in response to 10 mM sucrose. n = 3–4. Data are presented as mean ± SEM. One-way ANOVA with Tukey HSD test was used for comparison with control flies. (TIF) [file pgen.1009527.s011.tif]

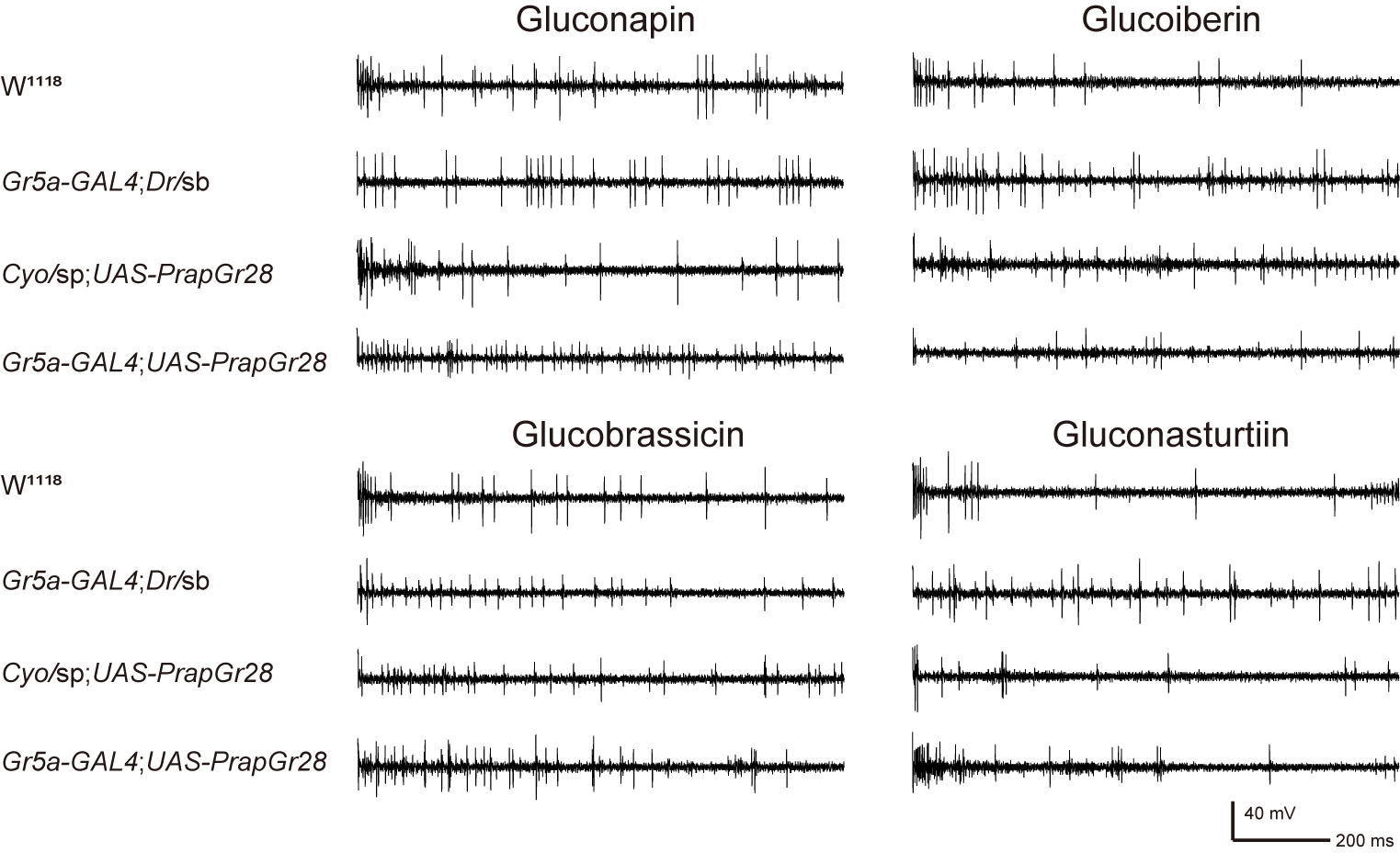

Supplement: S12 Fig — (TIF) [file pgen.1009527.s012.tif]

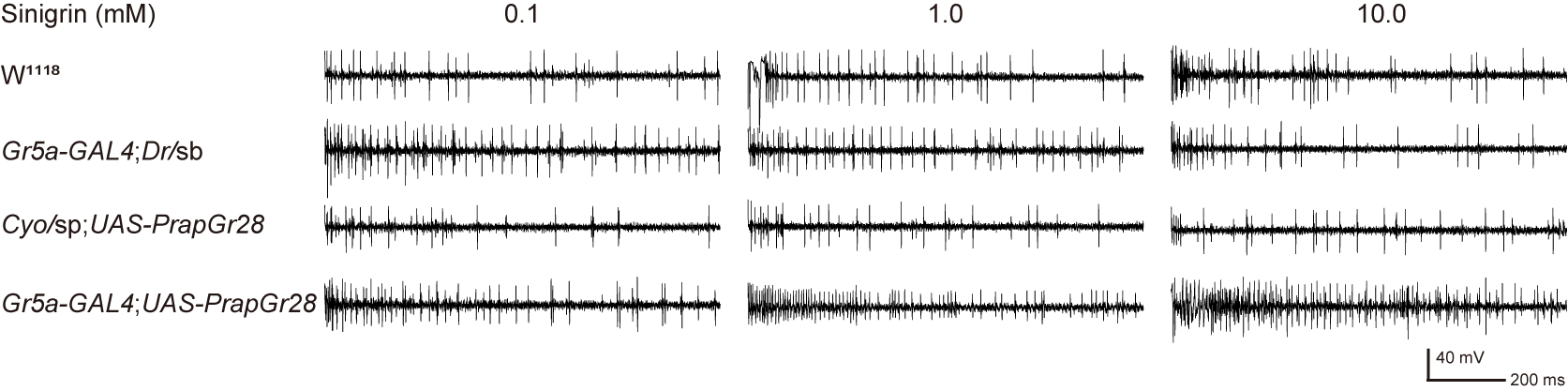

Supplement: S13 Fig — Example of response of L-type sensilla in the w1118, Gr5a-GAL4;Dr/sb, Cyo/sp;UAS-PrapGr28, and Gr5a-GAL4;UAS-PrapGr28 fly lines to different concentrations of sinigrin. (TIF) [file pgen.1009527.s013.tif]

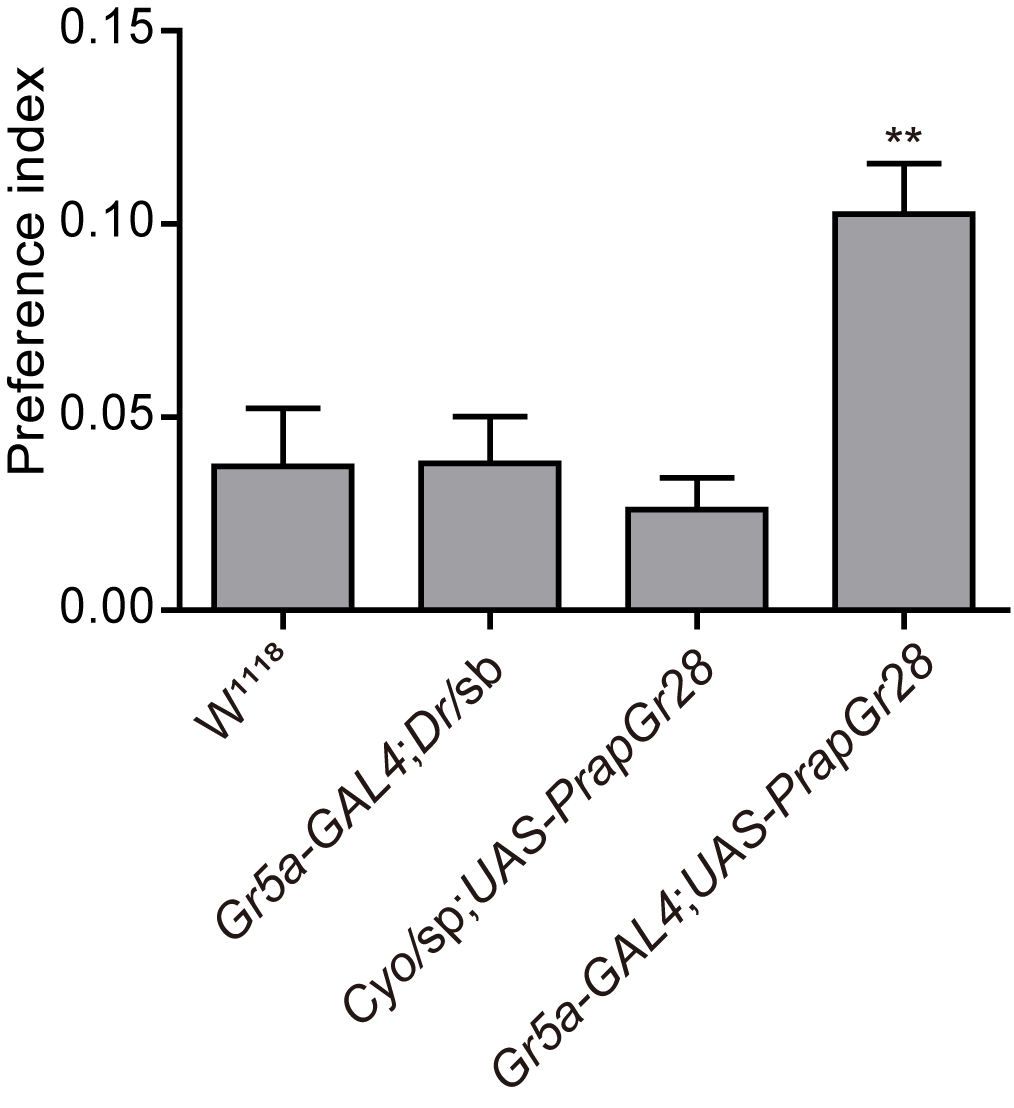

Supplement: S14 Fig — The presence of PrapGr28 reduced the aversive behavior to 10 mM sinigrin in the Gr5a-GAL4;UAS-PrapGr28 line. The w1118, Gr5a-GAL4;Dr/sb, and Cyo/sp;UAS-PrapGr28 fly lines were used as control lines. n = 10–11. Forty to fifty flies were used for each replicate. Data are presented as mean ± SEM. One-way ANOVA with Tukey HSD test was used. ** P < 0.01, compared with control flies. (TIF) [file pgen.1009527.s014.tif]

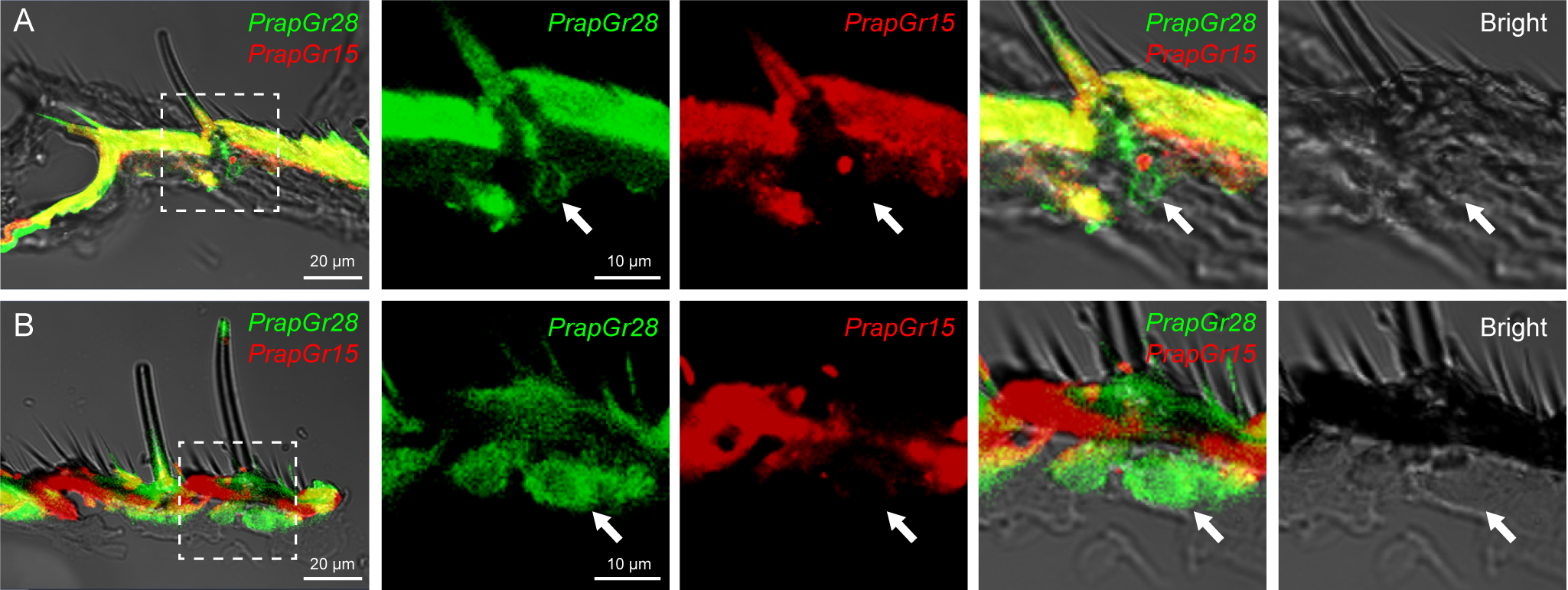

Supplement: S15 Fig — Co-expression patterns of PrapGr28 and PrapGr15 in female (A) and male (B) P. rapae adult foreleg tarsi. PrapGr28 antisense RNA probe was biotin-labeled and visualized by green fluorescence. PrapGr15 antisense RNA probe was digoxigenin-labeled and visualized by red fluorescence. The dashed frame areas are enlarged and shown on the right. Arrows show labelled somata with probes synthesized from targeted genes. Bright-field images are presented as references. (TIF) [file pgen.1009527.s015.tif]

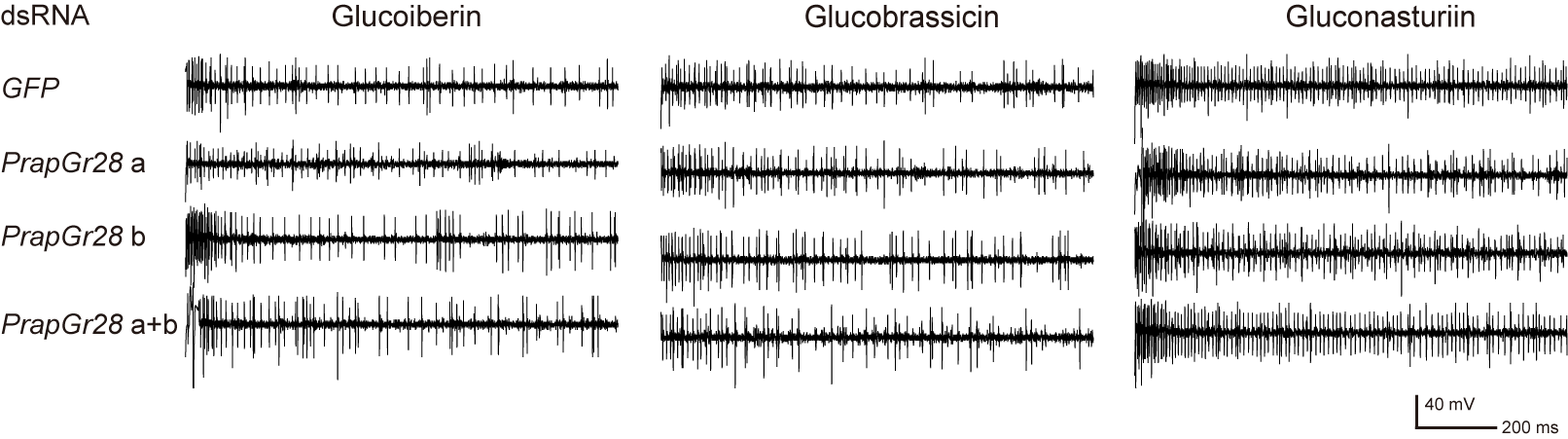

Supplement: S16 Fig — (TIF) [file pgen.1009527.s016.tif]

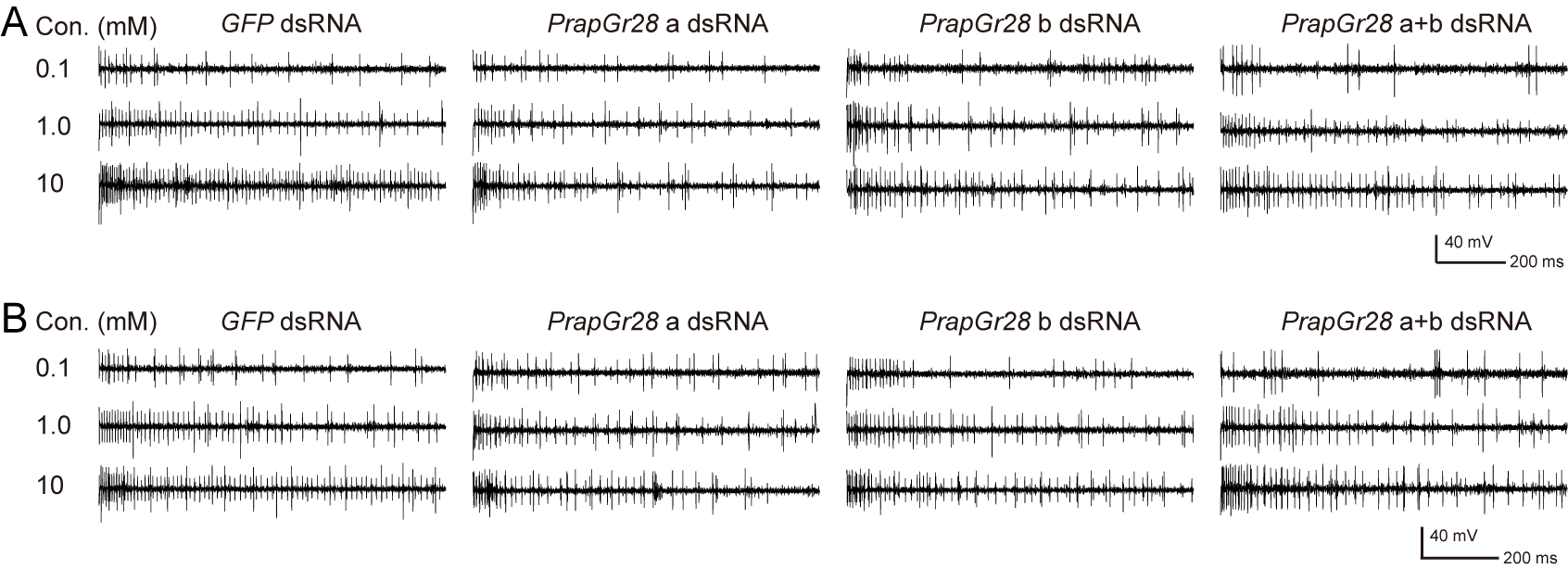

Supplement: S17 Fig — (TIF) [file pgen.1009527.s017.tif]

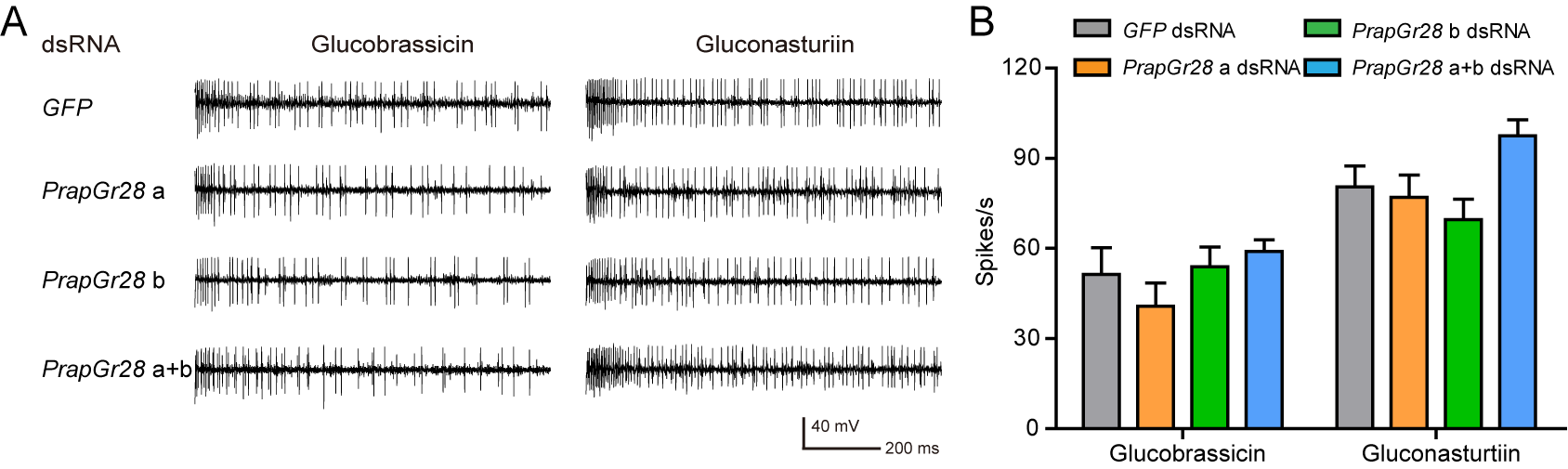

Supplement: S18 Fig — (A) Typical electrophysiological responses and (B) spike frequencies of lateral tarsal sensilla in response to 10 mM glucobrassicin (n = 4–8) and gluconasturtiin (n = 6–10). Data are presented as mean ± SEM. One-way ANOVA with Tukey HSD test was used for comparison with the control of GFP dsRNA. (TIF) [file pgen.1009527.s018.tif]
