# Supplementary material for: Identification of a gustatory receptor tuned to sinigrin in the cabbage butterfly Pieris rapae
Source: PLoS Genet. 2021 Jul 15;17(7):e1009527. doi: 10.1371/journal.pgen.1009527 (PMC8282186; doi:10.1371/journal.pgen.1009527)
Supplement: S1 Table — (DOCX) [file pgen.1009527.s019.docx]

**S1 Table. Candidate GRs in *P. rapae*.**

| **Name** | **GenBank No.** | **ORF (bp)** | **ORF (aa)** | **TC^a^** | **Identity** | **Top blastx hit** | **Evalue** |
| --- | --- | --- | --- | --- | --- | --- | --- |
| PrapGr1 | MT792867 | 1380 | 459 | Yes | 85% | AIG51907.1 | 0 |
| PrapGr2 | MT792868 | 537 | 179 | No | 91% | XP_004932263.1 | 4E-124 |
| PrapGr3 | MT792869 | 1101 | 366 | Yes | 77% | AIG51909.1 | 0 |
| PrapGr4 | MT792870 | 1362 | 453 | Yes | 42% | XP_011560065.1 | 1E-106 |
| PrapGr5 | MT792871 | 1023 | 341 | No | 50% | AIG51911.1 | 4E-121 |
| PrapGr6 | MT792872 | 984 | 328 | No | 68% | XP_011560061.1 | 2E-155 |
| PrapGr7 | MT792873 | 1314 | 437 | Yes | 53% | CAD31947.1 | 5E-145 |
| PrapGr8 | MT792874 | 1065 | 354 | Yes | 26% | DAA06383.1 | 3E-23 |
| PrapGr9 | MT792875 | 330 | 109 | Yes | 34% | DAA06383.1 | 1E-15 |
| PrapGr10 | MT792876 | 1023 | 340 | Yes | 26% | DAA06383.1 | 6E-27 |
| PrapGr11 | MT792877 | 1017 | 338 | Yes | 28% | DAA06383.1 | 3E-32 |
| PrapGr12 | MT792878 | 561 | 186 | Yes | 55% | EHJ73518.1 | 2E-57 |
| PrapGr13 | MT792879 | 438 | 146 | No | 29% | DAA06383.1 | 2E-15 |
| PrapGr14 | MT792880 | 384 | 127 | Yes | 35% | DAA06383.1 | 5E-22 |
| PrapGr15 | MT792881 | 1071 | 356 | Yes | 22% | DAA06381.1 | 4E-13 |
| PrapGr16 | MT792882 | 1050 | 349 | Yes | 33% | DAA06384.1 | 3E-15 |
| PrapGr17 | MT792883 | 1038 | 345 | Yes | 23% | DAA06384.1 | 3E-14 |
| PrapGr18 | MT792884 | 1041 | 346 | Yes | 26% | DAA06384.1 | 2E-15 |
| PrapGr19 | MT792885 | 1056 | 351 | Yes | 35% | NP_001124346.1 | 2E-71 |
| PrapGr20 | MT792886 | 387 | 129 | No | 39% | NP_001124346.1 | 1E-25 |
| PrapGr21 | MT792887 | 645 | 215 | No | 30% | DAA06388.1 | 5E-23 |
| PrapGr22 | MT792888 | 1080 | 359 | Yes | 41% | NP_001233216.1 | 1E-92 |
| PrapGr23 | MT792889 | 363 | 120 | Yes | 81% | EHJ69979.1 | 5E-32 |
| PrapGr24 | MT792890 | 501 | 167 | No | 30% | AGR03744.1 | 7E-17 |
| PrapGr25 | MT792891 | 972 | 323 | Yes | 36% | DAA06389.1 | 1E-57 |
| PrapGr26 | MT792892 | 642 | 213 | Yes | 39% | DAA06389.1 | 9E-44 |
| PrapGr27 | MT792893 | 318 | 106 | No | 39% | DAA06389.1 | 1E-19 |
| PrapGr28 | MT792894 | 1200 | 399 | Yes | 36% | QHB15307.1 | 3E-67 |
| PrapGr29 | MT792895 | 456 | 151 | Yes | 33% | XP_012550856.1 | 1E-23 |
| PrapGr30 | MT792896 | 483 | 160 | Yes | 49% | XP_012550565.1 | 2E-44 |
| PrapGr31 | MT792897 | 561 | 186 | Yes | 26% | DAA06383.1 | 4E-22 |
| PrapGr32 | MT792898 | 363 | 120 | Yes | 41% | DAA06389.1 | 2E-26 |
| PrapGr33 | MT792899 | 699 | 232 | Yes | 31% | XP_011561237.1 | 1E-43 |

^a^, Termination codon.
