# Supplementary material for: Identification of a gustatory receptor tuned to sinigrin in the cabbage butterfly Pieris rapae
Source: PLoS Genet. 2021 Jul 15;17(7):e1009527. doi: 10.1371/journal.pgen.1009527 (PMC8282186; doi:10.1371/journal.pgen.1009527)
Supplement: S2 Table — (DOCX) [file pgen.1009527.s020.docx]

**S2 Table. Primers used in this study.**

| **Purposes** | **Primer Names** | **Sequences (5'–3')** |
| --- | --- | --- |
| **qRT-PCR** | |  |
| 1 | qPrapGr1-F | ATGAACTTGGATGGCTTCAC |
|  | qPrapGr1-R | ATCCTCGTCATCTTCCATGT |
| 2 | qPrapGr2-F | GAGTCTGTCGGAGTGTTTTC |
|  | qPrapGr2-R | TCTGACAGTGCACCGTATAT |
| 3 | qPrapGr3-F | TGAAATACTGAACGGATGGC |
|  | qPrapGr3-R | AATACCAATATCCGCCCAGA |
| 4 | qPrapGr4-F | GTAATAGCTCGCTTGTCAGG |
|  | qPrapGr4-R | TTCTGATTTTGGCATTCGGT |
| 5 | qPrapGr5-F | CGTGCTGCTATCAAATGG |
|  | qPrapGr5-R | TCCACCAAAGCCAATGTC |
| 6 | qPrapGr6-F | ATGTCAGATTCACCTGGTTGTC |
|  | qPrapGr6-R | GCCACTTTCTTGCCACTTG |
| 7 | qPrapGr7-F | GCTGGATGGTCAAGGGATA |
|  | qPrapGr7-R | TCTTCAAGTTGCGGTCAGG |
| 8 | qPrapGr8-F | CAACAGAGCTATAAAAGTCAAGATG |
|  | qPrapGr8-R | AACTAAGCTTGCTTTAATATTCTCG |
| 9 | qPrapGr9-F | TGGTCCACTGTTTATTGAGC |
|  | qPrapGr9-R | ATTCACAGTTACAATCCGCC |
| 10 | qPrapGr10-F | AAATCAGCCTCTTTAGTCGC |
|  | qPrapGr10-R | TCGTGACCACAACATCTCA |
| 11 | qPrapGr11-F | TATGATGCCCTGTCCGCTT |
|  | qPrapGr11-R | ACAACGCCCGATGAAGTTC |
| 12 | qPrapGr12-F | AGACGGTAAAGGAGGACAAA |
|  | qPrapGr12-R | GTAAACCCCAAAGCTGTCAT |
| 13 | qPrapGr13-F | ATCATTAAGGCTGGCATTCG |
|  | qPrapGr13-R | AGGACAATGTTCGGGTAGTT |
| 14 | qPrapGr14-F | TGTGCCAGGTATTTTCTCAG |
|  | qPrapGr14-R | CGAGGTCACTTGCCAATAAA |
| 15 | qPrapGr15-F | TTGCTTAAGTTAGACGGTCAAA |
|  | qPrapGr15-R | GATCCATTCAAACGTCAAACTC |
| 16 | qPrapGr16-F | TGTTGCTTCAGACAGTTCCA |
|  | qPrapGr16-R | GACGTAGGTGGTTAATAGAGCA |
| 17 | qPrapGr17-F | CGTGATAATGACAACCATCG |
|  | qPrapGr17-R | GCCAGCAAGCCATAAGAATA |
| 18 | qPrapGr18-F | GACTCCGATAAACAAGCCA |
|  | qPrapGr18-R | GCACAACAGAGATACCAGCA |
| 19 | qPrapGr19-F | AACAGATTTGTCACCAGCC |
|  | qPrapGr19-R | GGCGAATGTCATAAAGTGC |
| 20 | qPrapGr20-F | GCCGCGCAAAAGATTTTCTC |
|  | qPrapGr20-R | TGTGACGTAGATACCCTCGA |
| 21 | qPrapGr21-F | AAAATCATCCCCAACTCGTG |
|  | qPrapGr21-R | ACGCGTCTCAAAATCATTGT |
| 22 | qPrapGr22-F | GGGAAACAGAAAGCCATCT |
|  | qPrapGr22-R | CCTAAGCCCATAAGGACCTG |
| 23 | qPrapGr23-F | ACTGTGTTGTTATTGAGGCC |
|  | qPrapGr23-R | TCTACTATGTCGTCTGCTGG |
| 24 | qPrapGr24-F | AATGCGGCACCAGTCTTA |
|  | qPrapGr24-R | GGGTCAACTCTACCATCAGAA |
| 25 | qPrapGr25-F | CACCCCAAACGAAAACGGAA |
|  | qPrapGr25-R | CCCACCAAACTGTAGGCGAT |
| 26 | qPrapGr26-F | AATGATTGTGTGGTCTTCGT |
|  | qPrapGr26-R | TGGACAAGCATTCACAACTT |
| 27 | qPrapGr27-F | ACGAAATGTCACCCTTAACG |
|  | qPrapGr27-R | TGTTGAAATCAGCGTTAGGA |
| 28 | qPrapGr28-F | CGATTATTGGGCTTGGCT |
|  | qPrapGr28-R | TTTACGAAGAGAGTCTGTTGCG |
| 29 | qPrapGr29-F | AATTCACCTACTCAGACGGT |
|  | qPrapGr29-R | GAAATACGACTCGGCAATCA |
| 30 | qPrapGr30-F | GACGGCACACTTATGATAGC |
|  | qPrapGr30-R | AAATGACAGATTGCGAGCTT |
| 31 | qPrapGr31-F | ACGAGAGACTCTGAGAAAGC |
|  | qPrapGr31-R | GGCCGATACAAACACCAATA |
| 32 | qPrapGr32-F | TGGTGCACAACTGAAGTATG |
|  | qPrapGr32-R | CCTTTAAAGTTGTCGCCTGA |
| 33 | qPrapGr33-F | CATACCAGCAACACTTCGAA |
|  | qPrapGr33-R | TGTTGTACCTCCATCATCCA |
| 34 | qEF1-F | GACAAACCGTGGCTGTAGGAG |
|  | qEF1-R | TTGCCGCCCTTGGTAGC |
| ***Xenopus* oocytes expression** | | |
| 1 | XPrapGr15-F | ATGTTAAAAACATCAAAATTCCTTAAA |
|  | XPrapGr15-R | TTATCCAAACGCATGAGTAAATTGTACAGT |
| 2 | XPrapGr28-F | ATGATATTGAAACATCGATTGCCG |
|  | XPrapGr28-R | TTAATTAACATTTTGTAATAATAT |
| 3 | XPrapGr15-*EcoR* Ⅰ-F | CGGAATTC*GCCACC*ATGTTAAAAACATCAAAATTCCTTAAA |
|  | XPrapGr15-*Xho* Ⅰ-R | CCCTCGAGTTATCCAAACGCATGAGTAAATTGTACAGT |
| 4 | XPrapGr28-*Stu* Ⅰ-F | CGAGGCCT*GCCACC*ATGATATTGAAACATCGATTGCCG |
|  | XPrapGr28-*Xho* Ⅰ-R | CCCTCGAGTTAATTAACATTTTGTAATAATAT |
| **Transgenic *Drosophila*** | |  |
| 1 | PrapGr28-F | same as XPrapGr28 primers |
|  | PrapGr28-R | same as XPrapGr28 primers |
| 2 | PrapGr28-RT-F | GATACACAGATGCAGTACAAGT |
|  | PrapGr28-RT-R | AGCATTTTACTCCCTCAACATT |
| 3 | Tub-F | TTAACGTGGATCTGACTGAGTT |
|  | Tub-R | GTACATCAGATCGAACTTGTGG |
| ***in situ hybridization*** | |  |
| 1 | iPrapGr28-F | TCCATTGATGCTTCGACATTAT |
|  | iPrapGr28-R | TTTTAAATCATCACTGGGCTCA |
| 2 | iPrapGr15-F | ATTGTCCTTTGTTGGTGGTAAT |
|  | iPrapGr15-R | ACGCATGAGTAAATTGTACAGT |
| **dsRNA** |  |  |
| 1 | GFP-F | ACCCTGAAATTCATCTGCAC |
|  | GFP-R | GGAGTGTTCTGCTGGTAATG |
| 2 | PrapGr28 a-F | ATGATATTGAAACATCGATTGCCG |
|  | PrapGr28 a-R | ACGAAGAGAGTCTGCTGCGTC |
| 3 | PrapGr28 b-F | ATCTAATGCATATAGACTCTCG |
|  | PrapGr28 b-R | TTAATTAACATTTTGTAATAATAT |
| 4 | T7GFP-F | TAATACGACTCACTATAGGACCCTGAAATTCATCTGCAC |
|  | T7GFP-R | TAATACGACTCACTATAGGGGAGTGTTCTGCTGGTAATG |
| 5 | T7PrapGr28 a-F | TAATACGACTCACTATAGGATGATATTGAAACATCGATTGCCG |
|  | T7PrapGr28 a-R | TAATACGACTCACTATAGGACGAAGAGAGTCTGCTGCGTC |
| 6 | T7PrapGr28 b-F | TAATACGACTCACTATAGGATCTAATGCATATAGACTCTCG |
|  | T7PrapGr28 b-R | TAATACGACTCACTATAGGTTAATTAACATTTTGTAATAATAT |
| 7 | qPrapGr28-F | TCCGGTGGTGTCTACATTAA |
|  | qPrapGr28-R | AGGTTGGACATGCAGAATTC |
| 8 | qEF1-F | same as qRT-PCR primers |
|  | qEF2-R | same as qRT-PCR primers |

F: forward primer; R: reverse primer. qRT-PCR (q), *in situ* hybridization (i), *Xenopus* oocytes expression (X) and T7 polymerase promoter (T7). Letters with underline indicates restriction enzyme sites. Italic letters represent the Kozak sequence which is used to enhance the translation efficiency.
